# Questionable research practices in student final theses – Prevalence, attitudes, and the role of the supervisor’s perceived attitudes

**DOI:** 10.1371/journal.pone.0203470

**Published:** 2018-08-30

**Authors:** Anand Krishna, Sebastian M. Peter

**Affiliations:** 1 Department of Motivational and Emotional Psychology, Julius-Maximilians-Universität, Würzburg, Germany; 2 Department of Social Psychology, Julius-Maximilians-Universität, Würzburg, Germany; Tilburg University, NETHERLANDS

## Abstract

Although questionable research practices (QRPs) and p-hacking have received attention in recent years, little research has focused on their prevalence and acceptance in students. Students are the researchers of the future and will represent the field in the future. Therefore, they should not be learning to use and accept QRPs, which would reduce their ability to produce and evaluate meaningful research. 207 psychology students and fresh graduates provided self-report data on the prevalence and predictors of QRPs. Attitudes towards QRPs, belief that significant results constitute better science or lead to better grades, motivation, and stress levels were predictors. Furthermore, we assessed perceived supervisor attitudes towards QRPs as an important predictive factor. The results were in line with estimates of QRP prevalence from academia. The best predictor of QRP use was students’ QRP attitudes. Perceived supervisor attitudes exerted both a direct and indirect effect via student attitudes. Motivation to write a good thesis was a protective factor, whereas stress had no effect. Students in this sample did not subscribe to beliefs that significant results were better for science or their grades. Such beliefs further did not impact QRP attitudes or use in this sample. Finally, students engaged in more QRPs pertaining to reporting and analysis than those pertaining to study design. We conclude that supervisors have an important function in shaping students’ attitudes towards QRPs and can improve their research practices by motivating them well. Furthermore, this research provides some impetus towards identifying predictors of QRP use in academia.

## Introduction

In recent years, consternation in the scientific community has been growing due to the potentially widespread use of *questionable research practices* (QRPs). Different institutions define QRPs in various ways, but many broader definitions refer to a subset of behaviors involving either fabrication or falsification (intentional distortion) of scientific data or results. In many cases, fabrication of data is viewed separately from QRPs, but both fall under the larger umbrella of practices likely to impair scientific progress ([1]; for a more complete discussion, see [2]). QRPs in a more narrow sense are usually equated with the exploitation of particular degrees of freedom in research design and data analysis in order to produce a specific (biased) result [3,4]. In recent years, the prevalence of such practices has been examined both on the level of academic science in general (e.g. [5]) and on the level of individual disciplines such as medical research (e.g. [6]), academic economics [7], and psychology [8–10]. In the latter field, the discussion of QRPs has generally centered on specific forms of distortion based on data collection rules and incomplete reporting of statistical results [3], exemplified by the survey by John and colleagues [9]. Although there is some disagreement on the actual prevalence of such practices [8], John and colleagues’ work found alarmingly high rates of research psychologists who reported having engaged in QRPs at least once in their career. For most of the ten individual behaviors surveyed, more than a quarter of the participants admitted to having done so in the past; only two of the behaviors (claiming that results were independent of demographic variables without a firm basis and falsifying data) showed prevalence below 10%. Even the more conservative estimates obtained by Fiedler & Schwarz [8] showed admission rates of over 10% for seven of the ten behaviors. Further research has also provided evidence for QRP use in psychology beyond self-report responses to survey, including unsolicited disclosures [11], application of algorithmic statistic-checking tools to published papers [12] and comparisons of online survey contents to published reports of that research [13,14]. Although estimates for individual QRPs vary between these studies, it seems clear that there is a significant issue with their use in academic psychology today.

These findings have been key in pushing the field towards increased transparency in data collection and reporting [15]. The drive towards replication in particular, including large-scale replication attempts such as the Many Labs projects [16,17] and the Open Science Collaboration [18], has sparked much discussion and raised awareness of the issues associated with QRPs [19–22]. Furthermore, several academic journals have revised their submission guidelines in order to increase the likelihood that researchers will avoid QRPs (e.g. [23]). Although researchers seem somewhat pessimistic about the field in general [22], there are clear signs that psychology is adapting its norms and standards to address the issue of QRPs [20,22].

However, although many studies have focused on the state of the art in academic psychology, few have yet addressed how that state informs students of the field. Even if QRP use may be declining and awareness of the issues surrounding it rising in academic psychology [24], this does not necessarily imply that teaching practices have changed. Although some authors and professional organizations have proposed general avenues of improvement [25–28] and others have advocated specific approaches to incorporating issues concerning QRPs into academic teaching programs [29–33], it remains unclear whether the field’s increased awareness is reflected in what students of psychology learn about the subject. To our knowledge, no research to date has examined even the prevalence of and attitudes towards QRP use among students, much less their understanding of how QRPs may affect scientific endeavors. Therefore, acquiring information about these basic questions is an important first step towards assessing whether future psychologists will be better capable of dealing with the challenges presented by QRPs. In addition, comparing student QRP use with QRP use by academic professionals may shed some light on what role academic socialization plays in QRP use: do academics learn about QRPs and stop using them as they develop or does academic culture play a role in fostering QRP use [34,35]? Although such questions can only be properly addressed by longitudinal studies that assess these variables, understanding the general status of QRPs in student work is a necessary starting point for such research.

Students generally do not conduct much independent research before finishing their primary degrees. However, for many students, their thesis work is the exception and a major milestone in their academic development. In many cases, such theses comprise an empirical study, supervised by a doctoral candidate or higher-level professional academic. As the most important empirical work for many students, the final thesis is a vital opportunity for them to acquire and apply practical research skills. It is also the point at which most students will first have the chance to employ QRPs. Finally, students typically write their thesis near the end of their studies, at a time when they have already learned most of what the course will teach them. Therefore, practices and attitudes during the thesis are an ideal predictor of students’ final opinions on QRPs. In addition, theses are of particular interest for other reasons. Although many empirical Bachelor’s and Master’s theses may never see scientific publication in their original form, their data may well form the basis of further work, whether by the supervisor or the student, or may be included in longer publications that comprise multiple studies. Furthermore and more importantly, such theses are the ultimate expression of the standards of scientific rigor that students learn and internalize over the period of their courses. For both those who continue academic work and those who pursue other avenues after completing their degree, the quality of their thesis work can be understood as an important indicator of how well they are capable of executing scientific research. Therefore, understanding QRP prevalence in student theses is vital to predicting how the field of psychology may develop both inside and outside academia.

### Causes of QRP Use in student work

QRPs are clearly detrimental to the pursuit of scientific knowledge [3], which raises the question why they are so prevalent. Some research exists addressing this question for academics. For example, some scientists might engage in QRPs due to a lack of knowledge about what constitutes a QRP and what effect this might have on the interpretation of the findings [36,37]. However, insofar as scientists know a particular practice to constitute a QRP, an important contributing factor to the choice to engage in it may be pressure to successfully publish papers in order to further one’s career (“publish-or-perish”, [1,33,38–40]). Specifically, the competitive requirement to publish may decrease adherence to scientific ideals [41] and be used as a justification for misconduct, including QRPs [36].

However, these causes of QRPs must be reevaluated when examining student work. Most students do not write their theses with the proximal goal of publication in an academic journal, but in order to fulfill a course requirement with (ideally) a good grade. The relevant outcome is not “publish-or-perish”, but rather “pass-or-fail”. Although students are likely to be motivated to achieve a good grade in a somewhat similar way that scientists are to publish their findings in prestigious journals, this is not directly comparable. Specifically, student grading is not as dependent on fitting a thematic “scope” as publications in focused journals are, especially as thesis topics are typically agreed upon with the supervisor prior to writing. In addition, publication space is limited in prestigious journals, so scientists may be in direct competition with one another, but this is not true in the majority of student theses–a good grade on a thesis is likely to be less dependent on the quality of theses by other students. Aside from the incentive structures involved, students are supervised by thesis advisors who are better versed in research methodology and statistics, potentially lowering the impact of a lack of statistical skills on the part of the student. In addition, students may expect their supervisors to be highly familiar with data patterns in their paradigm in general (as the supervisor likely has much direct experience with the paradigm) and with the specific data collected for the thesis (as the supervisor was likely directly involved). Especially the latter point might lead students to believe that any questionable statistical practices they engage in will be likely to be detected. For academics, it is by no means clear that an anonymous reviewer would have the expertise and conscientiousness to detect statistical anomalies. For these reasons, students are not necessarily subject to the same influences to engage in QRPs as full-fledged academic researchers.

So which alternative variables might play a role for the student population? The most proximal variable that should determine QRP use is attitudes towards said use. If students believe QRPs are actually good practice, they should be more likely to engage in them [42]. However, due to the influence wielded by supervisors in thesis work, the impact of their attitudes and beliefs on QRP use is also relevant. There are essentially two possible effects supervisor QRP attitudes may exert on QRP use in theses: first, supervisors may directly influence the thesis study, for example by providing the student with existing paradigms or analysis scripts or by coordinating data collection. Second, as students are learning about scientific practice as they work on their thesis, the attitudes of their supervisors may play a particularly important role in shaping their own. If a student perceives their supervisor as endorsing a specific research practice, this should influence their own evaluation of that practice, even if the practice is not actually useful or adequate. Students might apply an expert heuristic or change their attitudes to be more in line with a powerful supervisor or one they like [43,44]. Furthermore, supervisors may function as models for learning [45]. Theories of leadership [46] also predict that the way a supervisor thinks and acts has an important impact on what their subordinates will learn is appropriate behavior. Therefore, supervisor attitudes may affect QRP use in student work either directly or indirectly.

Furthermore, students are, on the whole, motivated to achieve as good a grade as possible on their final thesis [47,48]. Given this specific goal, they are likely to engage in practices which are conducive towards getting a good grade. Although ideally good scientific practice should be the primary determinant of grading of student theses, this may not match students’ perceptions. Some supervisors may explicitly [49] or implicitly express a belief that they consider students who can “produce effects” to be more competent than those who cannot, which in turn may lead students to believe that the statistical significance of their findings will affect their grade. If they believe either that significant findings are scientifically more valuable than nonsignificant ones, that good scientific work inevitably produces significant results or that their supervisor will reward them more for significant results, students may engage in more QRPs in order to ensure a better grade. They may also have more positive attitudes towards QRPs in general, either because said QRPs lead to ‘more valuable’ significant results or because their supervisors’ perceived preference for significant results also upvalues methods that lead to such results, for example via persuasion [43] or rationalization [50]. Furthermore, students may be under a large amount of stress when writing their thesis, which may in turn lead them to cut corners in their designs or analyses [33]. Several QRPs may not only bias the interpretation of the data, but also reduce the amount of work necessary, especially those that involve incomplete reporting. For example, if a student feels that an outlier in the data should be eliminated to present a clearer ‘story’, but is under pressure to finish writing, they may decide to eliminate the outlier without reporting it in order to save the time and effort involved in formulating a justification or the criteria for elimination. Similarly, failing to mention certain dependent measures might often save time compared to describing them in the study design, listing their analyses and discussing their meaning for the hypotheses, which may in turn help in coping with stress. On the other hand, students who are intrinsically motivated to perform well in their theses might engage in fewer QRPs. Insofar as they are not solely motivated by external rewards such as grades, but also by interest in the topic, they should be more committed to finding valid results, which should reduce the risk of QRP use.

The ability of students to ‘p-hack’ or otherwise tweak their results may be more limited than that of a more senior researcher, however. Their influence over study designs and particulars of data collection may be constrained, depending on the degree of direct control their supervisor exerts on these aspects of the thesis. Existing studies of QRP prevalence list various individual practices that a researcher might engage in (e.g. [9]), some of which might be more accessible to students than others. Further work by Wicherts and colleagues [4] has attempted to systematize QRPs by the phase of a research project in which they take place. As the writers of theses, students are most likely to be able to freely engage in QRPs that concern *reporting and analysis* of data and designs (e.g. rounding of p-values, changing hypotheses, omitting experimental conditions), which are also more numerous in general [4]. This is due both to their greater direct involvement in reporting and the relatively simple adjustments required for reporting and analysis QRPs–it is far simpler to eliminate a condition in data analysis than it is to consistently analyze the data in intervals until a significant result is achieved. They should therefore be less likely to be able to engage in QRPs of what we will collectively refer to as *study design* (e.g. stopping data collection as soon as a finding is significant), which correspond to QRPs in the phases of hypothesizing, design and collection in Wicherts et al.’s checklist. Accordingly, the effects of their attitudes, experienced stress and motivation to write their theses may depend on the type of QRP. This aspect of student work is important, as it reflects to some degree typical structures of academic collaborations among professional researchers: often, different individual researchers may handle study planning, data collection, analysis and reporting for a given collaborative project. If predictors for QRP use vary between these facets, this may shed some light on what characteristics are particularly important for a data analyst as opposed to for an author of a paper, for example.

This study aims to address three important questions: first, it seeks to provide the first data on the prevalence of QRPs in student theses. Second, it investigates what attitudes and circumstances might induce students in particular to engage in QRPs. Third, it seeks to differentiate between different types of QRPs in order to evaluate whether they are affected by different predictor variables.

### Hypotheses

Although this study is primarily descriptive in nature, we can formulate several hypotheses that might explain student QRP use. First, students’ reported QRP prevalence should be higher for reporting and analysis QRPs than for study design QRPs.

*H1*: *Students report engaging in more reporting and analysis QRPs than study design QRPs*.

Second, students’ attitudes towards the QRPs themselves should be predictive of whether they engage in such QRPs [51]. Their motivation to write their thesis should reduce QRP use, while their stress level should increase QRP use.

*H2a*: *More positive student attitudes towards QRPs lead to greater reported QRP use*.
*H2b*: *Higher motivation to write the thesis leads to less reported QRP use*.
*H2c*: *Higher stress levels lead to greater reported QRP use*.

Third, students’ attitudes towards the meaning of statistically significant results should influence their QRP use indirectly via influencing their attitudes towards QRPs [51]. Furthermore, students’ beliefs about how their supervisors reward significant results should influence their QRP use directly and possibly indirectly via their attitudes towards QRPs. Finally, supervisor attitudes towards QRPs should also impact QRP use directly as well as indirectly via student attitudes.

*H3a*: *Students who believe that good science produces significant results should report engaging in in greater QRP use*. *This effect should be mediated by students’ QRP attitudes*.
*H3b*: *Students who believe that their supervisor will reward significant results better should report engaging in in greater QRP use*. *This effect should be mediated by students’ QRP attitudes*.
*H3c*: *More positive perceived supervisor attitudes towards QRPs should lead to greater reported QRP use*. *This effect should be mediated by students’ QRP attitudes*.

Finally, students are likely to have more direct control over QRPs that occur in reporting and analyzing data than over QRPs that occur during study design and data collection. Therefore, all of the above relationships should be stronger for reporting and analysis QRPs than for study design QRPs.

*H4*: *The type of QRP moderates all relationships predicted in the preceding hypotheses such that relationships are stronger for reporting and analysis QRPs than for study design QRPs*.

## Method

### Sample

Participants were recruited online via university and student mailing lists and social media sites in June and July of 2016. Only students who were currently writing a Bachelor’s or Master’s thesis in a psychology course at a German public university or had written such a thesis within the last two years were solicited. Participants had a chance to win one of four 40€ Amazon.com vouchers as compensation. A total of 252 participants responded to the questionnaire. Twelve participants did not indicate they were currently engaged in or had been engaged in a thesis in the last two years and were excluded. A further ten participants indicated their thesis had been at a private institution (*n* = 9) or outside Germany (*n* = 1) and so were excluded. Finally, 23 participants answered none of the QRP items. Thus, the final sample consisted of 207 participants (82.1% retention) nonrepresentatively distributed over 40 universities (see Table A in S1 Table). As participants were asked to indicate their university and the subdiscipline of their thesis, further demographic data were explicitly not collected as part of this study in order to ensure anonymity when collecting such potentially sensitive data. 46.4% of participants were currently working on their theses, the remainder had completed them within the last two years. 57.0% of participants referred to their Bachelor’s thesis in the survey, 42.0% to their Master’s thesis and 1.0% to their Diplom thesis (a German degree equivalent to a Master’s degree).

### Procedure

The study was conducted in accordance with the ethical guidelines of the Deutsche Gesellschaft für Psychologie (DGPs). As no negative impact on participants was expected that exceeded typical daily emotional experiences, no specific ethics approval was required under these guidelines. Participants provided informed consent on the first page of the survey by clicking continue. The study was conducted online using the SoSciSurvey platform as part of a longer questionnaire that assessed various aspects of students’ experiences when writing their final theses. Although many measures are not relevant to the current study, they will be listed for completeness. On average, participants spent about 12 minutes on the entire questionnaire. After initial questions on the type of thesis (current or past, empirical or not, Bachelor’s or Master’s degree), institution, and subfield of psychology, participants gave basic information about their semester and the period in which they had started and (if applicable) ended their thesis work. Next, they were asked the academic degree of their supervisor as well as how much time they and their supervisor spent on the thesis per week and several items on their perceived autonomy in doing so. Thereafter, participants completed the measure of stress, followed by the critical measure of motivation and further items on their supervisor’s perceived motivation and specific aspects of motivation. Next came the FIT questionnaire [52]. Thereafter, participants completed a measure of grade pressure and the measures of belief that good science leads to significant results and belief that the supervisor rewards significant results. Next, self-reported QRP use was assessed, followed by attitudes towards QRPs and perceived supervisor attitudes towards QRPs. Finally, participants were given the option to express any comments they had, thanked and the study ended.

### Measures

#### Predictors

All predictors were measured using scales generated specifically for this study. Table 1 shows all items and scale metrics. The stress scale items were scored on a 4-point Likert scale (1 “statement does not apply” to 4 “statement applies”), all other scales were scored on a 5-point Likert scale (1 “disagree” to 5 “agree”). Most scales achieved acceptable to good internal consistency. The motivation scale also achieves acceptable consistency if the reverse-coded item is removed (Cronbach’s α = .80), however, the results are not meaningfully affected by this change, so the scores from the entire scale are used in analyses.

**Table 1:** 

*Scale Items and Reliability for Predictors*		
Scale name	Items	Cronbach’s α	
Stress	I felt stressed while working on my final thesis. Working on my final thesis is a strain for me. I don’t find writing my final thesis particularly stressful. (rev.)	.82
Motivation	I am motivated to work on my final thesis. I find working on my final thesis exciting and interesting. Working on my final thesis is not particularly important for me. (rev.)	.62
Good science leads to significant results	If someone does good scientific work, they produce significant results. I believe that null effects are generally caused by sloppy experimental work. A null effect tells me that I did not do good work.	.65
Supervisor rewards significant results	My grade depends on whether my results are significant. If my thesis does not produce publishable results, I will get a worse grade. I think my supervisor grades significant results better than nonsignificant ones.	.81

*Note*. All items are translated. “(rev.)” indicates an item that was reverse-scored for the scale. The items shown were adapted into the past tense where applicable for participants who indicated their thesis was already complete. *N* = 207 for all scales.

#### QRP use and related attitudes

Table 2 shows all the QRPs that were evaluated in this study. Those assessed by Fiedler & Schwarz [8] were augmented by an item on HARKing (Hypothesizing After the Results are Known; [53]) that was somewhat broader than Fiedler & Schwarz’ formulation as well as by several encouraged practices. As we assumed that not all students would know whether all QRPs were negative, we decided to add several positive or at least neutral practices in order to prevent participants from assuming all items were negative and answering them in a blanket fashion. Furthermore, we felt that it might be of interest to examine the prevalence of these practices side-by-side with QRPs. Self-reported QRP use was assessed with the item “In my thesis work, I at least once engaged in…” with the possible responses yes, no, or don’t know/can’t judge. Student (“Please indicate to what degree you consider the following procedures or research practices scientifically sensible or problematic”) and perceived supervisor (“Please indicate–as far as you know–to what degree your supervisor considers the following procedures or research practices scientifically sensible or problematic“) attitudes towards QRPs were assessed on a 5-point Likert scale from 1 –sensible to 5 –problematic with an additional option “can’t judge”.

**Table 2:** 

*QRPs and Positive or Neutral Practices*	
Practice	Type
Failing to report all dependent measures that are relevant for a finding	Reporting and analysis QRP (Fiedler & Schwarz)
Failing to report all conditions that are relevant for a finding	Reporting and analysis QRP (Fiedler & Schwarz)
Rounding off p *v*alues (e.g. reporting a p value of .054 as .05)	Reporting and analysis QRP (Fiedler & Schwarz)
Selectively reporting studies regarding a specific finding that “worked”	Reporting and analysis QRP (Fiedler & Schwarz)
Deciding whether to exclude data after looking at the impact of doing so regarding a specific finding	Reporting and analysis QRP (Fiedler & Schwarz)
Claiming to have predicted an unexpected result	Reporting and analysis QRP (Fiedler & Schwarz)
Claiming that results are unaffected by demographic variables (e.g. gender) although one is actually unsure (or knows that they do)	Reporting and analysis QRP (Fiedler & Schwarz)
Falsifying data	Reporting and analysis QRP (Fiedler & Schwarz)
Changing or formulating new hypotheses after analyzing the data	Reporting and analysis QRP (self-generated)
Collecting more data after seeing whether results were significant in order to render non-significant results significant	Study design QRP (Fiedler & Schwarz)
Stopping data collection after achieving the desired result concerning a specific finding	Study design QRP (Fiedler & Schwarz)
Conducting a power analysis	Positive or neutral practice (self-generated)
Reporting effect sizes	Positive or neutral practice (self-generated)
Utilizing a sequential analysis for planned early data collection stopping	Positive or neutral practice (self-generated)
Using Bayesian analysis	Positive or neutral practice (self-generated)

*Note*. All items are translated.

## Results

### Descriptive data

Table 3 shows the relative frequencies of the assessed practices separated by practice type from most frequent to least frequent. For the QRPs, between 75% and 85% of the participants gave a response of yes or no, while between 5% and 15% indicated they could not judge whether the QRP had been implemented. Of those who could judge, the majority of QRPs were practiced by less than 11%. The average self-admission rate was 9.3%. Only selective reporting of studies, data exclusion post-analysis and HARKing showed a prevalence of 15% or higher. These numbers are noticeably lower than the researcher self-report scores reported by John et al. [9], in line with Fiedler & Schwarz’ [8] expectations for their modification of the QRP items and with the fact that this survey assessed QRPs in one specific project rather than over a researcher’s working lifetime (see Table B in S2 Table for a direct comparison of prevalence estimates from both of these studies). Comparing our results to Fiedler & Schwarz’ estimated prevalence rates of individual QRPs, most QRPs have similar self-reported prevalences, but rounding off p values and deciding whether to exclude data post-analysis are noticeably more common in our sample (*Δcf* > 5%), while selectively reporting studies that “worked” is much more common (*Δcf* > 15%). It is unclear whether these differences are due to random sampling error, measurement error in Fiedler & Schwarz’ estimation of prevalence or to a systematic difference between our sample of German students and their sample of German researchers, but they apparently do not support the conclusion that students are employing fewer QRPs than their seniors. However, the largest difference, that for selectively reporting studies, must be interpreted with caution. Student theses generally do not comprise a series of studies from which the student may then pick which to report. Looking at the therefore surprisingly high prevalence of this QRP, it is possible that this question was interpreted differently by this sample than by that of Fiedler & Schwarz. For example, students might have interpreted this item as referring to their discussion of the relevant literature in their theory sections. Although it might be considered questionable to knowingly present a one-sided view of the scientific literature on a given topic as well, this is not comparable to Fiedler & Schwarz’ sample, who presumably understood the question as referring to selective reporting of their own experiments.

**Table 3:** 

*Relative Frequencies of Practices*			
Practice	*n* responding	of which “yes”	of total sample “don’t know”
Selectively reporting studies	159 (75.8%)	28.3%	13.0%
Deciding whether to exclude data after looking at the results	161 (77.8%)	15.5%	12.1%
Changing or formulating new hypotheses after analyzing the data	171 (82.6%)	15.0%	7.2%
Rounding off p *v*alues	163 (78.7%)	10.4%	11.6%
Claiming to have predicted an unexpected result	165 (79.7%)	10.3%	10.1%
Failing to report all relevant conditions	166 (80.2%)	7.7%	9.7%
Failing to report all relevant dependent measures	157 (75.8%)	5.8%	14.0%
Falsifying data	175 (84.5%)	2.9%	5.3%
Falsely claiming that results are unaffected by demographics	155 (74.9%)	2.6%	15.0%
Collecting more data in order to achieve significance	169 (81.6%)	2.4%	8.2%
Stopping data collection after achieving the desired result	173 (83.6%)	1.9%	6.3%
Reporting effect sizes	162 (78.3%)	69.1%	11.6%
Conducting a power analysis	150 (72.5%)	35.3%	16.9%
Using Bayesian analysis	119 (57.5%)	3.9%	32.4%
Utilizing sequential analysis	158 (76.3%)	1.0%	7.2%

*Note*. The first column shows the number (and percentage) of participants who responded either “yes” or “no”, the second shows the relative frequency of yes responses among such participants. The third column is the percentage of all 207 participants who responded “don’t know/can’t judge”; the shortfall to 100% is due to item nonresponses.

Table 4 shows how many participants reported engaging in multiple QRPs. If selective study reporting is omitted from analysis, it can be seen that the majority of participants reported engaging in no QRPs at all. More than 90% of participants engaged in less than three QRPs. Although there is significant room for improvement given that more than 40% of participants did engage in at least one QRP, these numbers indicate that student theses are not subject to very high concentrations of QRPs.

**Table 4:** 

*Concentration of Practices over Participants*		
Number of QRPs engaged in	Frequency (all)	Frequency (excluding selectively reporting studies)
0	80 (44.4%)	105 (58.3%)
1	60 (33.3%)	44 (24.4%)
2	20 (11.1%)	16 (8.9%)
3	9 (5.0%)	6 (3.3%)
4	6 (3.3%)	4 (2.2%)
5	1 (0.6%)	3 (1.7%)
6	2 (1.1%)	2 (1.1%)
7	2 (1.1%)	-

*Note*. The subsample is those participants who responded to at least one QRP item with “yes” or “no” (*n* = 180).

With regard to the positive/neutral practices, more than two thirds of responding participants indicate that they report effect sizes, which may reflect this practice becoming more normative. A third of the sample reported conducting a power analysis during their thesis work, which may indicate that power analysis is not yet a standard tool that students learn to apply (especially considering that 16.9% indicated that they did not know whether a power analysis had been conducted). This finding is somewhat surprising when contrasted with the large majority of academic researchers who reported having used power analyses in prior studies [22,28]. Finally, very few students make use of sequential analysis (e.g. [54]) or Bayesian analysis (e.g. [55])–for the latter, a noticeably high proportion (32.4%) are uncertain enough to be unable to answer.

Table 5 shows the participants’ own attitudes towards QRPs as well as their perception of their supervisors’ (see charts in S1 Bar Charts for information on attitude distribution). In general, there is a close correspondence between students’ attitudes and those they perceive their supervisors to have, although the correlations do not indicate that the items measured the same construct (average *r* = .41, see Table C in S3 Table). Participants hold a negative view of all QRPs and believe their supervisors to hold negative views of same. Descriptively, students appear to realize that QRPs are generally problematic for scientific research, indicating that their methodological training likely covers the issues associated with these practices. This is further bolstered by the low number of nonresponses on these items: apparently, the vast majority of participants felt certain enough to report an attitude on most of the QRPs. Furthermore, both reporting effect sizes and conducting power analyses are considered to be good practice by students. For Bayesian and sequential analysis, the number of nonresponses is far greater, indicating that students likely do not feel competent enough to make a judgment on these methods. Indeed, sequential analysis is perceived as slightly negative by those that do offer an attitude–this is likely reflective of students reading the description of “planned early data collection stopping” and equating it with a QRP. The fact that perceived supervisor attitudes follow the same pattern may imply that students perceive such attitudes as normative in psychology research.

**Table 5:** 

*Attitudes Towards QRPs*				
Practice	Participant Attitudes		Supervisor Attitudes	
	*n*	*M* (*SD*)	*n*	*M* (*SD*)
Selectively reporting studies	199	2.18 (.98)	121	2.03 (1.22)
Deciding whether to exclude data after looking at the results	189	1.96 (.94)	119	2.13 (1.25)
Changing or formulating new hypotheses after analyzing the data	201	1.92 (1.00)	140	1.97 (1.21)
Rounding off p *v*alues	196	2.02 (.97)	120	1.87 (1.19)
Claiming to have predicted an unexpected result	199	1.89 (.87)	134	1.78 (1.06)
Failing to report all relevant conditions	201	1.65 (.73)	129	1.66 (1.01)
Failing to report all relevant dependent measures	190	1.79 (.79)	127	1.75 (.97)
Falsifying data	202	1.08 (.47)	163	1.07 (.39)
Falsely claiming that results are unaffected by demographics	188	1.57 (.74)	126	1.52 (.86)
Collecting more data in order to achieve significance	199	2.21 (1.09)	123	2.34 (1.32)
Stopping data collection after achieving the desired result	199	2.04 (.96)	115	1.70 (.98)
Reporting effect sizes	192	4.61 (.74)	137	4.64 (.79)
Conducting a power analysis	160	4.47 (.65)	99	4.37 (.89)
Using Bayesian analysis	67	3.57 (1.02)	37	3.30^a^ (1.35)
Utilizing sequential analysis	100	2.23 (1.06)	59	1.97 (1.25)

*Note*. All attitudes are coded such that greater values indicate greater endorsement. All attitude values except those marked ^a^ differ significantly from the scale midpoint after Bonferroni correction (all *t* ≥ 4.55, all *p* ≤ .0001, all *d* ≥ .55).

### Confirmatory analyses

In preparation for the confirmatory tests, separate mean self-reported prevalence scores were calculated for reporting and analysis QRPs (deciding whether to exclude data after looking at the results, changing or formulating new hypotheses after analyzing the data, rounding off p *v*alues, claiming to have predicted an unexpected result, failing to report all relevant conditions, failing to report all relevant dependent measures, falsifying data, falsely claiming that results are unaffected by demographics) and study design QRPs (collecting more data in order to achieve significance, stopping data collection after achieving the desired result). If participants failed to respond to any QRP item, the mean prevalence was calculated without that score in order to maximize the sample size. Mean attitude scores for participants’ own and perceived supervisor attitudes were calculated analogously. The QRP ‘selectively reporting studies’ was not included in the confirmatory analyses due to reasons discussed above. It should be noted that inclusion of this QRP changed none of the results substantively. Descriptive data for all variables is shown in Table 6. In general, participants were somewhat motivated and somewhat stressed by their theses, but they did not endorse beliefs that significant results influenced their grade more or had more scientific meaning.

**Table 6:** 

*Descriptives for Confirmatory Analyses*		
Variable	*n*	*M* (*SD*)
Prevalence for reporting and analysis QRPs	179	.105 (.17)
Prevalence for study design QRPs	174	.026 (.12)
Participant attitude towards reporting and analysis QRPs	202	1.73 (.48)
Participant attitude towards study design QRPs	201	2.12 (.83)
Perceived supervisor attitude towards reporting and analysis QRPs	168	1.71 (.82)
Perceived supervisor attitude towards study design QRPs	136	2.09 (1.10)
Stress	207	3.22 (.76)
Motivation	207	3.73 (1.01)
Good science leads to significant results	207	1.45 (.59)
Supervisor rewards significant results	207	1.62 (.69)

A within-subjects t-test revealed that participants reported engaging in significantly more reporting and analysis QRPs than study design QRPs, *t*(172) = 5.63, *p* < .001, *d*_*z*_ = .43, thus confirming H1.

**Fig 1:**
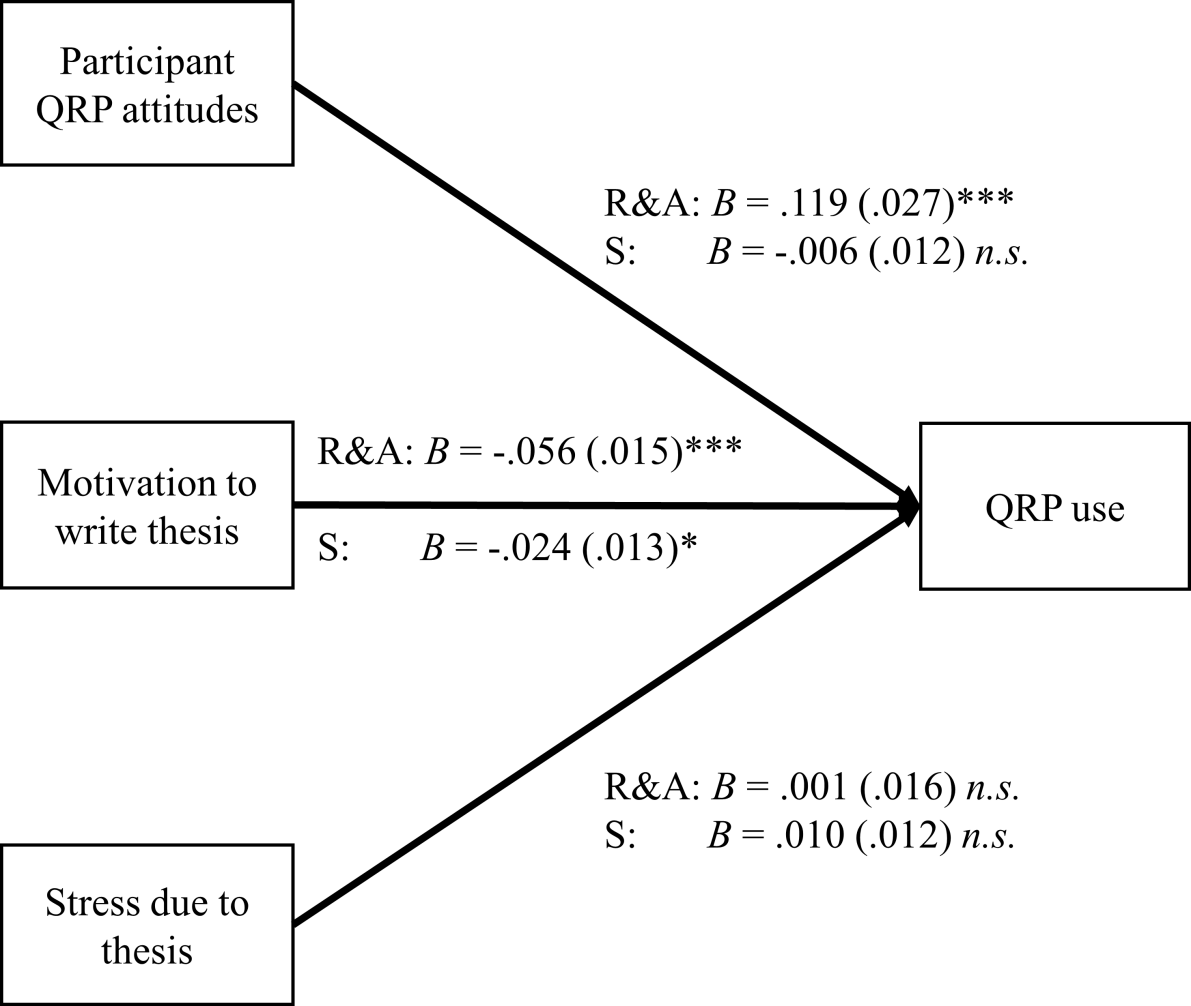
Regression weights for models predicting reporting and analysis (R&A) and study design (S) QRP use. Standard errors are given in parentheses. Asterisks denote statistical significance: *: *p* < .05, **: *p* < .01, ***: *p* < .001.

To evaluate H2, regression models were calculated that included stress, motivation, and participant QRP attitudes as predictors and self-reported QRP prevalence as the criterion (see Fig 1). One model each was calculated for reporting and analysis QRP prevalence and study design QRP prevalence with the respective attitude as the predictor. For reporting and analysis QRPs, the model achieved significance, *F*(3,171) = 16.58, *p* < .001, Adj. *R*^*2*^ = .212. Both participant attitudes (*p* < .001) and motivation (*p* < .001) achieved significance as predictors in the expected directions, although stress (*p* = .926) did not. For study design QRPs, the model did not achieve significance, *F*(3,167) = 1.94, *p* = .126, Adj. *R*^*2*^ = .016. In this model, only motivation (*p* = .044) achieved significance; neither participant attitudes (*p* = .606) nor stress (*p* = .458) were significant predictors. These results reflect support for H2a, partial support for H2b and no support for H2c. They also indicate that relationships between predictors and self-reported QRP prevalence are stronger for reporting and analysis QRPs, supporting H4.

**Fig 2:**
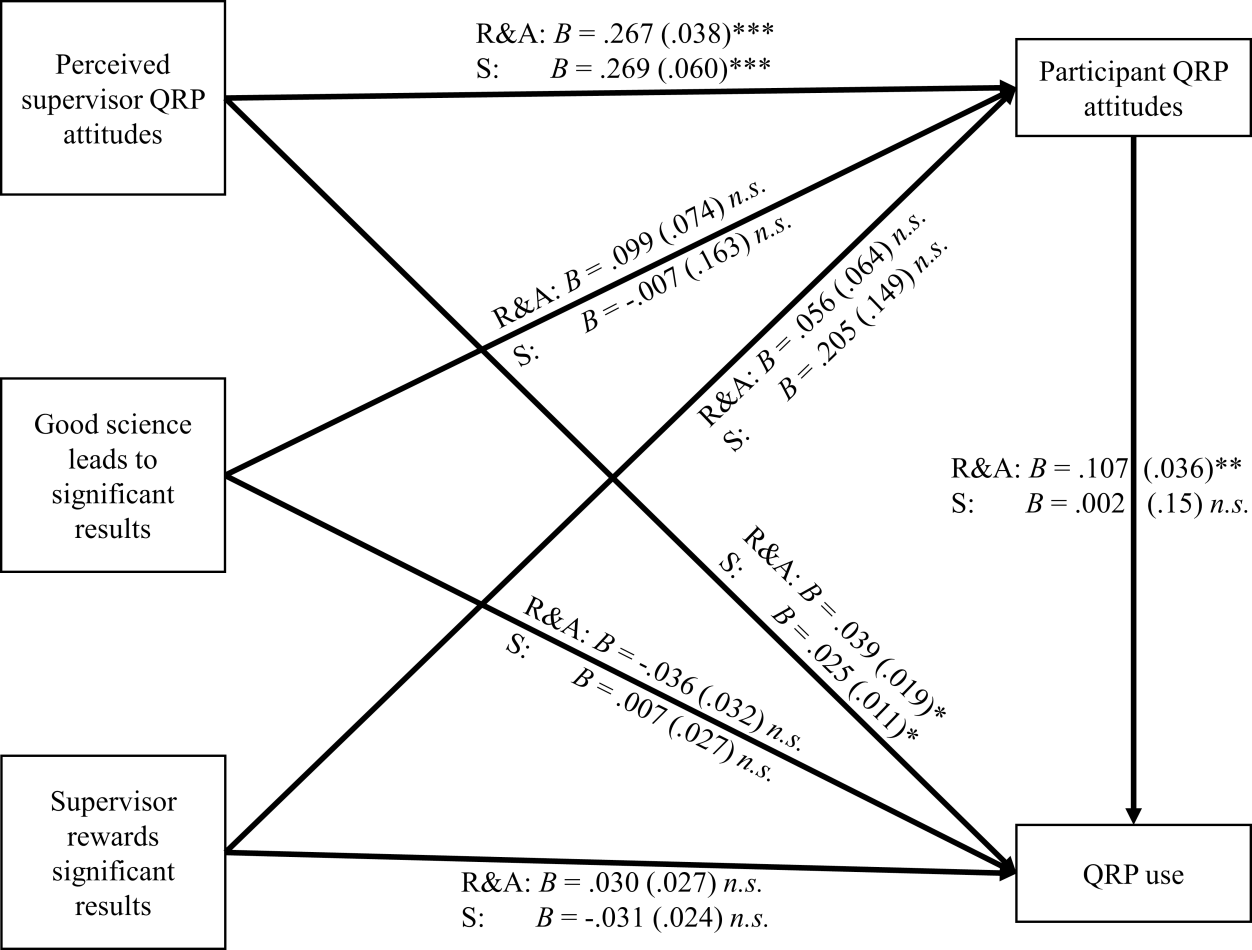
Regression weights for mediation models predicting self-reported reporting and analysis (R&A) and study design (S) QRP use. Standard errors are given in parentheses. Asterisks denote statistical significance: *: *p* < .05, **: *p* < .01, ***: *p* < .001.

To evaluate H3, mediation models were calculated using the PROCESS macro (v. 2.16,[56]) that included perceived supervisor attitudes, belief that good science leads to significant results, and belief that the supervisor rewards significant results as predictors, participant attitudes as a mediator and self-reported QRP prevalence as the criterion (see Fig 2, all reported confidence intervals for indirect effects are 95% CIs). Analogously to H2, separate models were calculated for the two QRP types. For the reporting and analysis QRP model, the model achieved significance, *F*(4,140) = 7.95, *p* < .001, *R*^*2*^ = .185. Perceived supervisor attitudes exerted both a direct (*p* = .040) and an indirect (*B* = .029, *SE* = .009, CI = [.013, .050]) effect on self-reported QRP prevalence. Belief that good science leads to significant results had neither a direct (*p* = .254) nor an indirect (*B* = .011, *SE* = .010, CI = [-.006, .035]) effect and the same held for belief that the supervisor rewards significant results (direct effect: *p* = .270; indirect effect: *B* = .006, *SE* = .008, CI = [-.006, .029]). For the study design QRP model, the model achieved marginal significance, *F*(4,115) = 2.13, *p* = .082, *R*^*2*^ = .069. In this model, perceived supervisor attitudes exerted a direct (*p* = .018), but no indirect (*B* = .001, *SE* = .005, CI = [-.010, .012]) effect on QRP prevalence. Again, neither belief that good science leads to significant results (direct effect: *p* = .791; indirect effect: *B* = .000, *SE* = .003, CI = [-.007, .006]) nor belief that the supervisor rewards significant results (direct effect: *p* = .211; indirect effect: *B* = .001, *SE* = .004, CI = [-.007, .012]) showed any significant results. These results do not support H3a or H3b, but show some support for H3c: the effect of perceived supervisor attitudes is partially mediated by participant attitudes, albeit only for reporting and analysis QRPs. This pattern of results also supports H4: the mediated effect of perceived supervisor attitudes is smaller for study design QRPs than for reporting and analysis QRPs. Further examination of the mediation model shows that this is due to the weaker link between participant QRP attitudes and self-reported QRP use for study design QRPs.

## Discussion

This study sought to evaluate the prevalence of various QRPs in student thesis works in psychology from public universities in Germany. It also aimed to shed light on determining factors for self-reported QRP use, including participants’ attitudes towards QRPs, towards the dependency of grades on QRPs, and about the importance of significant results for good science as well as their perception of their supervisors’ attitudes towards QRPs. Finally, it aimed to differentiate between reporting and analysis QRPs and study design QRPs, the former of which students should be more free to engage in. The results were generally in line with previous research on QRP prevalence [8], showing that although some QRPs are reported as being practiced by around 10% of students, the majority are comparatively rare. It is important to note that the results are not comparable with some of the prior research on QRPs [9,10]. John et al. [9] and Agnoli et al. [10] used formulations of QRP items that have been criticized as too broad and ambiguous [8,10] and which are likely to lead to larger estimates of QRP prevalence due to their broadness. On the other hand, the more precise Fiedler and Schwarz [8] formulations may underestimate the actual prevalence by giving participants opportunity to answer no even if they had engaged in a QRP because it did not fit the strict definition. The average self-admission rate of 9.3% was significantly lower than those found by other studies (John et al. [9]: 29.9%, Fiedler & Schwarz [8]: 25.5%, Agnoli et al. [10]: 27.3%), but this is to be expected, as this study measured an incidence over one project (the thesis) while the others measured lifetime incidence over all studies. Results were instead similar to the prevalence estimates from Fiedler & Schwarz [8]. However, this self-admission rate is likely underestimating the true rate of QRP use in theses. Furthermore, participants showed negative attitudes towards all QRPs and positive attitudes towards some recommended practices (such as reporting effect sizes). Potentially toxic attitudes about significant results being more indicative of good science as well as the perception that significant results would be rewarded by good grades were not endorsed by the sample. Finally, supervisors were also seen as condemning QRPs and encouraging good practice. Consistent with predictions, students reported engaging in more analysis and reporting QRPs than study design QRPs. Both their attitudes towards QRPs (positively) and their motivation to write their thesis (negatively) predicted self-reported QRP use, although their stress levels did not. In general, students’ beliefs about the necessity of significant results for good science or good grades did not impact their QRP use or attitudes, but their perception of their supervisors’ attitudes affected both. These data have several implications for teaching practice as well as for further QRP research.

### Implications for teaching practice

Although some research has indicated that there is a degree of pessimism in academic psychology with regard to QRP use [22], the results of this study are at least somewhat heartening. QRP use does not appear very widespread among German students according to their own reports (although this result may not generalize to all students [57]). Indeed, the majority of participants reported having engaged in none of the applicable QRPs from the list assessed and a further quarter only engaged in one. Although QRPs are generally likely to harm the evidentiary value of research, the greatest problems occur when multiple such practices are applied in order to p-hack data [3]. Furthermore, students are clearly able to identify QRPs as problematic. One might therefore conclude that although there is certainly room for improvement, students’ education seems quite adequate. However, it is interesting to note a few further protective factors. Beyond students’ endorsement of QRPs, two further predictors emerged as influential: the perceived attitude of the supervisor and the student’s motivation to write a good thesis.

It is clear that supervisors play an important role in mentoring students and teaching them vital skills [58–60]. However, this study’s results are consistent with the conclusion that they also strongly influence students’ practical understanding of methods they have already learned. Students have several sources of information from which they may infer their supervisors’ opinions on methodological practices, from direct information transmission in teaching to affective undertones when discussing specific issues. It is possible or even likely that students did not always know their supervisors’ specific attitude towards a given QRP, but inferred said attitude from the transmission of more general information about open science and methodological practices, thereby feeling confident enough to make a judgement instead of nonresponding. Although our measure of perceived supervisor attitude was unable to pinpoint the precise source of their perceptions, these perceptions nevertheless affected their reported QRP use. The effect supervisors’ perceived attitudes had on students’ reported QRP use was partially mediated by student attitudes. This pattern can be interpreted in terms of two parallel influences: first, supervisors’ expressed attitudes influence students’ attitudes and thereby their actions, and second, supervisors occasionally directly influence student research practices without necessarily modifying the students’ view. The former influence is only visible for reporting and analysis QRPs in this data, whereas the latter affects all relevant QRPs. This underlines the importance of both adequate methodological training during a course and proper reinforcement of the acquired methods during the final thesis. Methodology lecturers need not assume that their teachings will be suborned by actual practice in the final thesis, as students’ attitudes may at least partially resist QRP use by their supervisors. On the other hand, thesis supervisors must be aware that the attitudes they express towards QRPs may influence students even though those students have already studied methodology. Given that a majority of students will not continue in fields where they perform empirical studies subject to methodological scrutiny [61], their knowledge of how to conduct such research will likely remain stable after the end of their studies, making it vitally important that supervisors take the issue of QRPs seriously when mentoring their students. Importantly, the lack of an indirect effect of supervisor attitudes on self-reported QRP use for study design QRPs does not ameliorate this issue, as the analysis showed the effect on student attitudes remained similar.

Of course, this study followed a cross-sectional design, raising the question of whether the actual causality between the variables is in line with this interpretation of the data. As the questions were necessarily retrospective and no manipulation of the predictors was possible, alternative causal models may obtain. First, it is possible that participants inferred their supervisors’ and their own attitudes from the presence or absence of a given QRP in their work. However, even if this were the case, the main conclusion still holds that supervisors must take care not to tacitly endorse QRPs. Either QRP use is not the causal variable or QRP use biases attitudes post-hoc, in which case supervisors must actively prevent QRP use to prevent problematic attitudes that will likely cause more QRP use in the future [51]. The third possibility, that students inferred QRP use and supervisor attitudes from their own attitudes towards QRPs, is unlikely in the current study. If a student was uncertain about the former two variables, they had the option to indicate they did not know or could not judge. It is unclear why a student would believe that they could reliably judge their supervisor’s attitude or the presence of a specific practice in their thesis based on their own attitude if they also had the option of indicating their uncertainty. Furthermore, the correlations between students’ own attitudes and their perceptions of their supervisors’ were mostly of medium-to-high magnitude (.3 < *r* < .5), rather than of .7 or higher, which indicates that these perceptions were being influenced by other factors than their own attitudes. It should be underlined, however, that the mediation models presented in this study cannot prove causation. Indeed, reanalysis of this relationship with participant attitudes as the predictor and perceived supervisor attitudes as the mediator also produced a significant indirect effect, implying that these data cannot rule out alternative causal models [56]. However, it is a plausible interpretation with consequences for supervisor behavior that supervisors’ expression of opinions about QRPs may influence their students’ attitudes. As QRP attitudes are a reflection of students’ methodological competence going forwards, the supervisors’ projecting a critical attitude towards QRPs remains important.

A further protective factor against QRP use was the students’ motivation to write their thesis. Particularly motivated students reported applying fewer QRPs in their studies, both in reporting and analysis and during study design. For reporting and analysis, this may reflect increased conscientiousness as a result of higher motivation, such that students are sufficiently motivated to take the time to do the extra analyses and writing that may be associated with transparent reporting. For study design, the question arises how student motivation influences an aspect of their thesis work over which they might have less control. One possibility might be that high motivation is associated with increased likelihood of exerting control over study design. Being involved in all aspects of a study, including its initial design, should produce a greater feeling of meaningfulness through increased task identity [62]. If so, highly motivated students might be highly motivated because they have influence in study design, while this increased motivation in turn leads them to make greater efforts to adhere to scientific standards. These results underline the importance of motivating students to apply themselves to their final theses from an educational standpoint. Highly motivated students report using fewer QRPs, which likely increases the quality of their work. Importantly, motivation is an aspect that educators can influence significantly when it comes to final theses [59,60,63,64]. Therefore, according to our data, supervisors can improve student outcomes with regard to QRPs the most by fostering motivation in their students and by monitoring their own expression of QRP-relevant attitudes.

An interesting proviso that might apply to these results is the role of the student’s beliefs in moderating the protective effects of motivation. If students believe that good science leads to significant results or that they need significant results to get good grades, this might reduce or even reverse the protective effect of motivation. Highly motivated students should be particularly likely to use QRPs in order to p-hack to the degree that they believe significant results are good. Although our exploratory post-hoc analyses to investigate this proposed relationship produced no clear evidence bolstering or falsifying it (see Analysis A in S1 Exploratory Analysis), this may be due to the lack of variance in these beliefs in our sample.

It is also possible that students who used fewer QRPs were more motivated as a result. To the degree that students condemn QRPs, engaging in them is likely aversive. A student forced to use a QRP (whether by their supervisor or some other circumstance) would therefore likely be less motivated to work on their thesis. Although such an account is not consistent with our finding that student QRP attitudes are closely related to students’ reported QRP use, we cannot rule out this possibility. Even so, the implication would then be that supervisors should not try to cut corners and encourage students to engage in QRPs, as this will likely negatively impact their motivation to work. This only bolsters the conclusion that supervisors must take care not to appear accepting of QRP use or they risk negative outcomes for students. Finally, it is possible that other variables we did not observe in our study may explain the effect of motivation on QRP use, such as student ability–more able students might be both more motivated and less likely to use QRPs–or broader attitudes towards science, such as general endorsement of the scientific method. Future studies might focus on these predictors, both in a general sense as determinants of motivations and in their specific role in affecting QRP use. However, as motivation is both easily assessed and a good target for intervention, we believe that these data offer a good starting point for practical measures to prevent student QRP use by increasing motivation.

### Implications for academic psychology

Even though academic psychologists’ usage of QRPs is most likely primarily determined by factors specific to an academic research environment [35,65,66], this study has several implications for their work as well. First and most obviously, this study provides some insight into the state in which academics begin their careers today. Although more research is needed to understand how precisely their socialization as scientists will affect their QRP usage, this study provides some insight into how they will initially approach the topic. The conclusions these data point to are somewhat encouraging: even though their self-reported QRP prevalence is similar to that of more established researchers, psychologists are unlikely to endorse QRP use at the beginning of their careers. Therefore, the likelihood is that the environment in a group composed of young researchers would be conducive to ethical behavior [67], reducing the likelihood of QRP use. Whether this in principle good environment can be maintained depends on other factors, however. Motyl and colleagues [22] note that they find no difference in QRP use between early and late career stages. Together with our results, this may indicate that early-career psychological scientists do not *like* QRPs, but may still feel pressured to *use* them. If so, it underlines that the proximal causes of this felt pressure are the most important targets for change in the field.

However, the effect that supervisors have on their students’ attitudes also holds important implications for how PhD candidates may be influenced by their thesis advisors. Although several researchers have postulated that mentoring quality is an important factor in QRP use [68–71], little systematic research has actually evaluated this link. Wright and colleagues [72] examined case files of the US Office of Research Integrity and concluded that supervisors often neglect their duties to educate their students about proper standards of research, whereas Ripley and colleagues [73] concluded that supervisors’ role in teaching research ethics was active, but insufficient. Both of these studies adopted a deficit model of mentoring’s effect on QRP use, seeing the major issue as lack of effort or responsibility on the side of the mentor. To our knowledge, our study is the first that examines pathways by which even active supervision may sometimes promote QRP usage. We provide quantitative evidence that in an environment where supervisors are perceived as endorsing QRPs, students are also more likely to do so. Although our study was conducted with undergraduate and Master’s students, it seems likely that similar relationships would hold for PhD students and their thesis advisors.

A further gain of our study for understanding QRP use in academic environments comes from our attempt to relate QRP endorsement to precursor attitudes about statistical significance. Although some QRP use in academic environments may be due to ignorance of their negative impact [36], some researchers may hold strong beliefs about the need for statistically significant results to further their careers (analogous to students wanting same to get better grades) which may drive them to apply QRPs even against their better judgment. Others might be convinced that the scientific value of a significant result outweighs the problems associated with using QRPs based on a flawed understanding of statistical inference. Our study is the first to assess such attitudes and evaluate their links towards QRP endorsement and reported use. The students in our sample generally did not hold such attitudes, so this study’s ability to assess their impact may have been lessened. However, although our data do not show any impact of beliefs about the importance of significant results for scientific progress or of better rewards for significant results on self-reported QRP use or attitudes, the effect sizes particularly in the reporting and analysis model are descriptively in the expected direction and neither the p-values nor the distribution of such beliefs in our sample allow us to draw the conclusion that such an impact is falsified. Therefore, this study underlines the need to more precisely identify such precursor attitudes while also providing initial suggestions for what they might be.

Finally, we differentiate between different kinds of QRPs according to in what stage of the experimental process they occur [4]. In many academic projects, researchers collaborate with one another. In some collaborations, technical skills, workload or logistical constraints may determine in which aspects of a project a researcher is involved. Some researchers may be concerned only with study design aspects, particularly if the study requires specialized measurement tools or programming software, whereas others might be involved only in specialized analyses or in the writing of the resulting research report. In most collaborations, therefore, an individual’s propensity to engage in QRPs is likely to depend on their freedom to do so without scrutiny. Our study provides evidence that in assessing the impact of problematic attitudes towards QRPs on their use, it is important to consider such structural variables. In the albeit somewhat lopsided collaborations between students and supervisors observed in this study, students were expected to have less influence on study design and more on reporting and analysis. Bearing this idea out, student-level predictors such as attitudes towards QRPs and motivation had less impact on self-reported study design QRP use, while the direct influence of perceived supervisor attitudes remained. It seems likely that similar dynamics might hold in collaborations between professional academics. Therefore, our data show the relevance of not just researchers’ general attitudes towards QRPs, but also their role in a given project to their likelihood to employ them. In addition, our data also underline that supervision alone is likely insufficient to prevent QRP use. Supervisors may play an important role in whether students endorse or engage in QRPs, but they cannot themselves guarantee that students will not do so on their own. In a similar manner, junior collaboration partners may not always be able to prevent QRP use by their seniors, as can be seen in the direct influence exerted by supervisor attitudes on self-reported QRP use. Placing the onus solely on either supervision or on individual researchers’ behavior might be oversimplifying the cause of QRP use.

When drawing conclusions for the wider academic field from these data, it is important to bear in mind the differences between our student sample and professional researchers. Students are likely to be considerably less free in their experimental decision-making than most professional researchers might be assumed to be. Time constraints for the thesis as well as organizational constraints such as access to participant pools may limit students’ options and in some courses, they may not even be expected to implement some aspects of their study autonomously (such as programming experiments or performing analyses). Although these differences may seem to limit the generalizability of these results, it must be noted that many of these constraints may well also apply to early-career researchers in particular with only slight variations. Early-career researchers are often placed under time pressure by their funding limits and may face stiff competition for organizational resources such as lab space. Furthermore, many early-career researchers might require more advanced colleagues’ or their PhD thesis advisors’ assistance with specific technical aspects of their work and in consequence allow said colleagues or advisors to dictate specific aspects of their designs or analyses through authority. Although the experiences of students in their final theses therefore may not generalize unreservedly to academic professionals, they can still draw attention to the role of such constraints and may allow conclusions depending on relevant analogous variables in professional practice.

In addition, if the constraints students operate under with their theses do indeed change the processes leading to their QRP use, it begs the question why their self-reported QRP prevalence is so strikingly similar to that of professional researchers (e.g. [8]). Although it is possible that the underlying reasons for students’ QRP use are very different than those for researchers, it seems more likely that students either respond in an at least somewhat similar fashion to researchers or that researchers are in fact directly impacting student work. As an example for the latter case, for those decisions students are not free to make in their theses, their supervisors would have to be the ones conducting QRPs. Under these circumstances, the results of our study would have to be reinterpreted as the effect of supervisor QRP use on student attitudes: students who report having engaged in QRPs (due to their supervisors’ influence) presumably endorse them more post-hoc. In this case, our data speak strongly to the need to avoid forcing students into committing QRPs, which can certainly also be generalized to early-career researchers, who may operate under a similar lack of autonomy with regard to some aspects of their work. Our correlational design does not allow a reliable inference as to which causal direction holds, but the existence of a relationship between supervisor influence and student attitudes is clear. Further research is necessary to evaluate whether students’ (and possibly young researchers’) QRP attitudes are more impacted by being induced to use QRPs or by the influence of their supervisors’ attitudes.

Beyond the constraints experienced by students, however, the incentives vary between the groups: students are concerned with passing their courses and getting good grades, where researchers typically require publications. Although publications may also be seen on a gradient from high-impact to low-impact journals, each submission of a research paper is essentially an all-or-nothing scenario in which one either passes or fails, which may render significant results (which increase the chance of publication [38]) subjectively more important for the outcome. Furthermore, a thesis is a one-time task which may be perceived as disproportionately important by the student, whereas research submissions are regular occurrences for many academics, possibly affecting motivation. Although these differences surely impact whether the relationships demonstrated in this paper hold for academics, particularly with regard to significance beliefs and motivation, it may still be argued that academics’ experiences as students are the major determinant of how they enter the academic world. Additional pressures and influences in scientific work notwithstanding, identifying factors that affect how an early-career researcher views scientific practice may illuminate possible sources of resilience against falling into QRPs. A related issue that bears more scrutiny in future research is the acceptance and application of open science standards such as data sharing, more precise statistical reporting and pre-registrations, each of which have been discussed as countermeasures to widespread QRP use (e.g. [3,27]). Research has shown that psychologists are skeptical of regulations to enforce such measures [24], but there have already been attempts and recommendations to integrate these contents into teaching programs (e.g. [74]). As noted above, our data cannot show whether students are influenced more by their supervisors’ attitudes towards QRPs or by their inference of those attitudes from the supervisors’ general stance towards open science. The effectiveness of these initiatives in shaping young researchers’ attitudes is therefore yet to be assessed, a gap which future research might address.

## Conclusion

Our study aimed to shed light on the prevalence of QRPs in student thesis work and identify several predictors thereof. The final thesis is an important indicator of what critical research skills a student has acquired over the course of their studies and may also serve as a basis for further academic work either by the student or their supervisor, so data on QRPs in this context is of significant relevance. Our results show that QRP use is not particularly widespread among students according to their own reports, with less than 20% indicating having engaged in more than one. Students also reported moderately to strongly negative attitudes towards QRPs and more positive attitudes towards recommended research practices such as power analyses. Highly motivated students engaged in fewer QRPs, which may imply that properly motivating students in their thesis work is important not just for their enjoyment, but also for the scientific quality of their work. In general, it seems that supervisors may be able to prevent QRPs both by fostering motivation and by conveying their own negative attitudes towards QRPs clearly, thereby ensuring students perceive them as such. Finally, the most relevant QRPs for students seem to be reporting and analysis QRPs, for which the discussed variables have the greatest impact. For wider academic practice, our data suggest that endorsement of QRPs by supervisors might also impact junior researchers negatively. Future research should examine broader attitudes that might predict QRP attitudes as well as differentiate between QRPs that occur at various points in the scientific process.

## Supporting information

S1 Table Table with distribution of participants over universities. (DOCX)

S2 Table Table comparing prevalence estimates from the current study with those from John et al. and Fiedler & Schwarz. (DOCX)

S3 Table Correlations between participants’ own attitudes and their perceived supervisor attitudes. (DOCX)

S1 Bar Charts Bar charts depicting frequency distributions of responses to individual QRP attitude items. (PDF)

S1 Exploratory Analysis Analysis report for tests of moderation of the protective effect of motivation by student beliefs about statistical significance. (PDF)
