# Supplementary material for: Questionable research practices in student final theses – Prevalence, attitudes, and the role of the supervisor’s perceived attitudes
Source: PLoS One. 2018 Aug 30;13(8):e0203470. doi: 10.1371/journal.pone.0203470 (PMC6117074; doi:10.1371/journal.pone.0203470)
Supplement: S1 Table — (DOCX) [file pone.0203470.s001.docx]

Table A

| *Universities of participating students* | | | |  |
| --- | --- | --- | --- | --- |
| University | *n* | % of  *N* = 207 |  | |
| Bamberg | 6 | 2.9% |  |  |
| Berlin (Free University) | 1 | 0.5% |  |  |
| Berlin (Humboldt University) | 4 | 1.9% |  |  |
| Bielefeld | 2 | 1.0% |  |  |
| Bochum | 2 | 1.0% |  |  |
| Bonn | 3 | 1.4% |  |  |
| Braunschweig | 2 | 1.0% |  |  |
| Bremen | 5 | 2.4% |  |  |
| Chemnitz | 1 | 0.5% |  |  |
| Darmstadt | 1 | 0.5% |  |  |
| Dortmund | 2 | 1.0% |  |  |
| Eichstätt-Ingolstadt | 4 | 1.9% |  |  |
| Erfurt | 1 | 0.5% |  |  |
| Erlangen-Nürnberg | 4 | 1.9% |  |  |
| Frankfurt am Main | 2 | 1.0% |  |  |
| Freiburg | 2 | 1.0% |  |  |
| Gießen | 4 | 1.9% |  |  |
| Göttingen | 8 | 3.9% |  |  |
| Hagen | 4 | 1.9% |  |  |
| Halle-Wittenberg | 1 | 0.5% |  |  |
| Heidelberg | 10 | 4.8% |  |  |
| Hildesheim | 1 | 0.5% |  |  |
| Jena | 1 | 0.5% |  |  |
| Kassel | 2 | 1.0% |  |  |
| Kiel | 1 | 0.5% |  |  |
| Koblenz-Landau | 1 | 0.5% |  |  |
| Köln | 1 | 0.5% |  |  |
| Konstanz | 2 | 1.0% |  |  |
| Magdeburg | 1 | 0.5% |  |  |
| Mainz | 2 | 1.0% |  |  |
| Marburg | 5 | 2.4% |  |  |
| Münster | 2 | 1.0% |  |  |
| Osnabrück | 14 | 6.8% |  |  |
| Regensburg | 6 | 2.9% |  |  |
| Saarland | 7 | 3.4% |  |  |
| Trier | 6 | 2.9% |  |  |
| Tübingen | 6 | 2.9% |  |  |
| Ulm | 3 | 1.4% |  |  |
| Wuppertal | 4 | 1.9% |  |  |
| Würzburg | 73 | 35.3% |  |  |
