# Supplementary material for: Questionable research practices in student final theses – Prevalence, attitudes, and the role of the supervisor’s perceived attitudes
Source: PLoS One. 2018 Aug 30;13(8):e0203470. doi: 10.1371/journal.pone.0203470 (PMC6117074; doi:10.1371/journal.pone.0203470)
Supplement: S2 Table — (DOCX) [file pone.0203470.s002.docx]

Table B

| *Relative frequencies of practices in comparison to John et al. (2012) and Fiedler & Schwarz (2016)* | | | |  |
| --- | --- | --- | --- | --- |
| Practice | Prevalence  Current study | Prevalence  John et al. | Prevalence  Fiedler & Schwarz |  |
| Selectively reporting studies | 28.3% | 67% | 10.9% |  |
| Deciding whether to exclude data after looking at the results | 15.5% | 62% | 7.7% |  |
| Rounding off p *v*alues | 10.4% | 39% | 3.9% |  |
| Claiming to have predicted an unexpected result | 10.3% | 54% | 10.1% |  |
| Failing to report all relevant conditions | 7.7% | 42% | 4.7% |  |
| Failing to report all relevant dependent measures | 5.8% | 78% | 7.8% |  |
| Falsifying data | 2.9% | 9% | 0.3% |  |
| Falsely claiming that results are unaffected by demographics | 2.6% | 13% | 0.6% |  |
| Collecting more data in order to achieve significance | 2.4% | 72% | 6.0% |  |
| Stopping data collection after achieving the desired result | 1.9% | 36% | 1.0% |  |
| *Note.* The first column shows the percentage of participants who responded either “yes” from those who responded either “yes” or “no”. The third column shows Fiedler & Schwarz’s prevalence estimates based on the proportion of yes responders multiplied by the average repetition rate reported in their study. | | | |  |
