# Supplementary material for: Questionable research practices in student final theses – Prevalence, attitudes, and the role of the supervisor’s perceived attitudes
Source: PLoS One. 2018 Aug 30;13(8):e0203470. doi: 10.1371/journal.pone.0203470 (PMC6117074; doi:10.1371/journal.pone.0203470)
Supplement: S3 Table — (DOCX) [file pone.0203470.s003.docx]

Table C

| *Correlations between participant and perceived supervisor attitudes* | | | |  |
| --- | --- | --- | --- | --- |
| Practice | *r* | *p* |  | |
| Selectively reporting studies | .44 | <.001 |  |  |
| Deciding whether to exclude data after looking at the results | .44 | <.001 |  |  |
| Changing or formulating new hypotheses after analyzing the data | .54 | <.001 |  |  |
| Rounding off p *v*alues | .74 | <.001 |  |  |
| Claiming to have predicted an unexpected result | .33 | <.001 |  |  |
| Failing to report all relevant conditions | .20 | .021 |  |  |
| Failing to report all relevant dependent measures | .40 | <.001 |  |  |
| Falsifying data | .07 | .368 |  |  |
| Falsely claiming that results are unaffected by demographics | .45 | <.001 |  |  |
| Collecting more data in order to achieve significance | .42 | <.001 |  |  |
| Stopping data collection after achieving the desired result | .52 | <.001 |  |  |
| Reporting effect sizes | .54 | <.001 |  |  |
| Conducting a power analysis | .65 | <.001 |  |  |
| Using Bayesian analysis | .16 | .310 |  |  |
| Utilizing sequential analysis | .80 | <.001 |  |  |
| Reporting QRPs | .43 | <.001 |  |  |
| Study Design QRPs | .39 | <.001 |  |  |
| All QRPs | .42 | <.001 |  |  |
| *Note.* Scores for “Reporting QRPs” and “All QRPs” are calculated without “Selectively reporting studies”. | | |  |  |
