# Supplementary figures and images for: Questionable research practices in student final theses – Prevalence, attitudes, and the role of the supervisor’s perceived attitudes
Source: PLoS One. 2018 Aug 30;13(8):e0203470. doi: 10.1371/journal.pone.0203470 (PMC6117074; doi:10.1371/journal.pone.0203470)

Bar charts for descriptive variables.

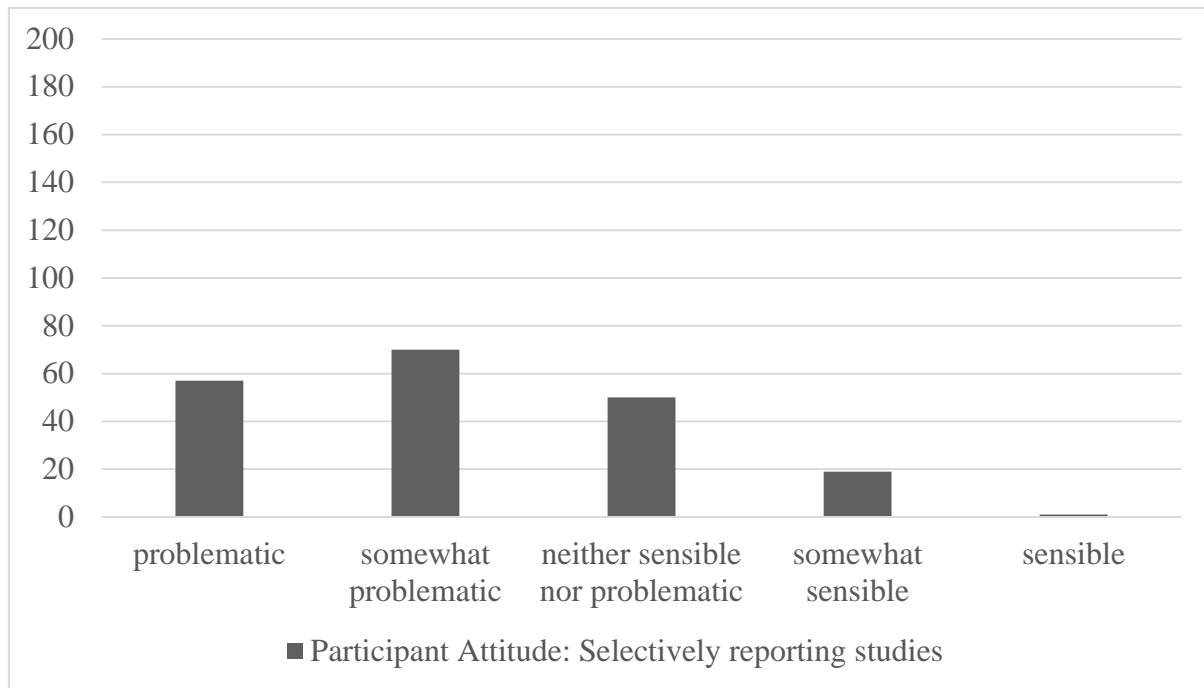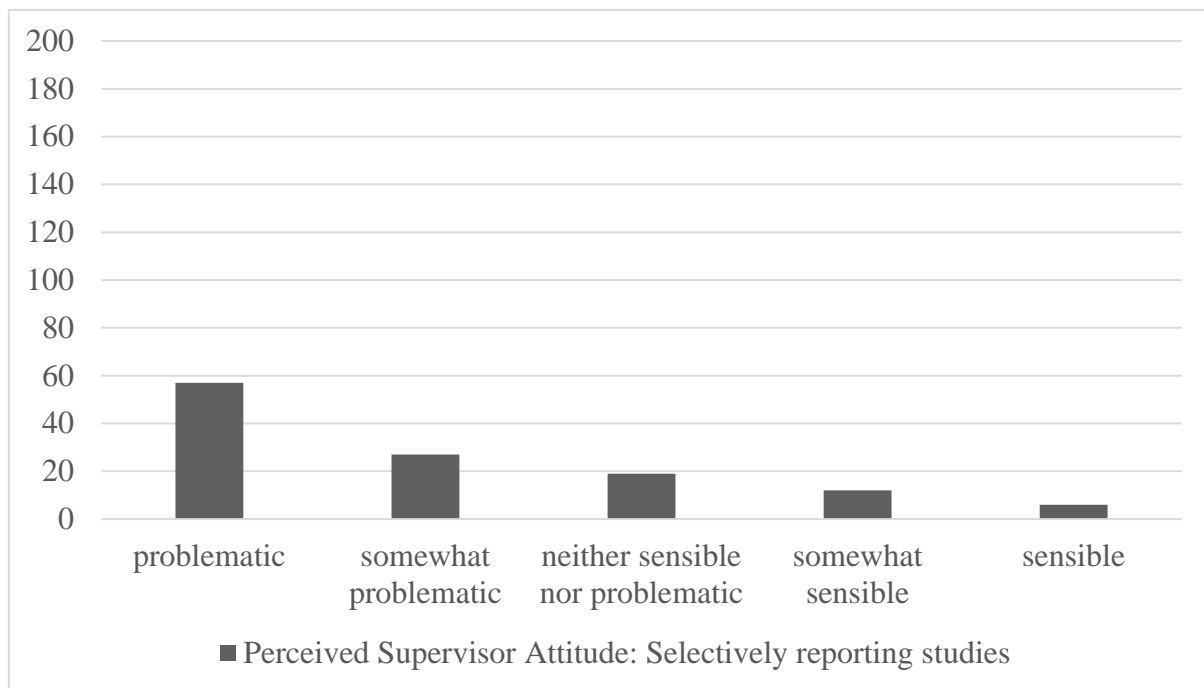

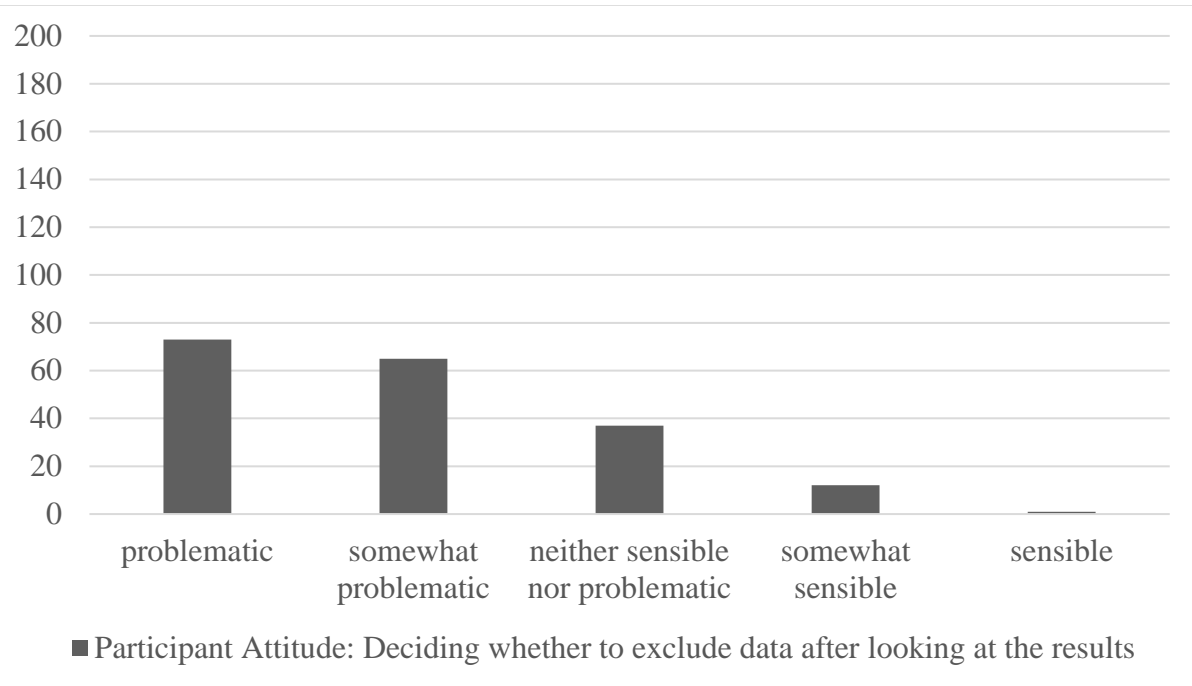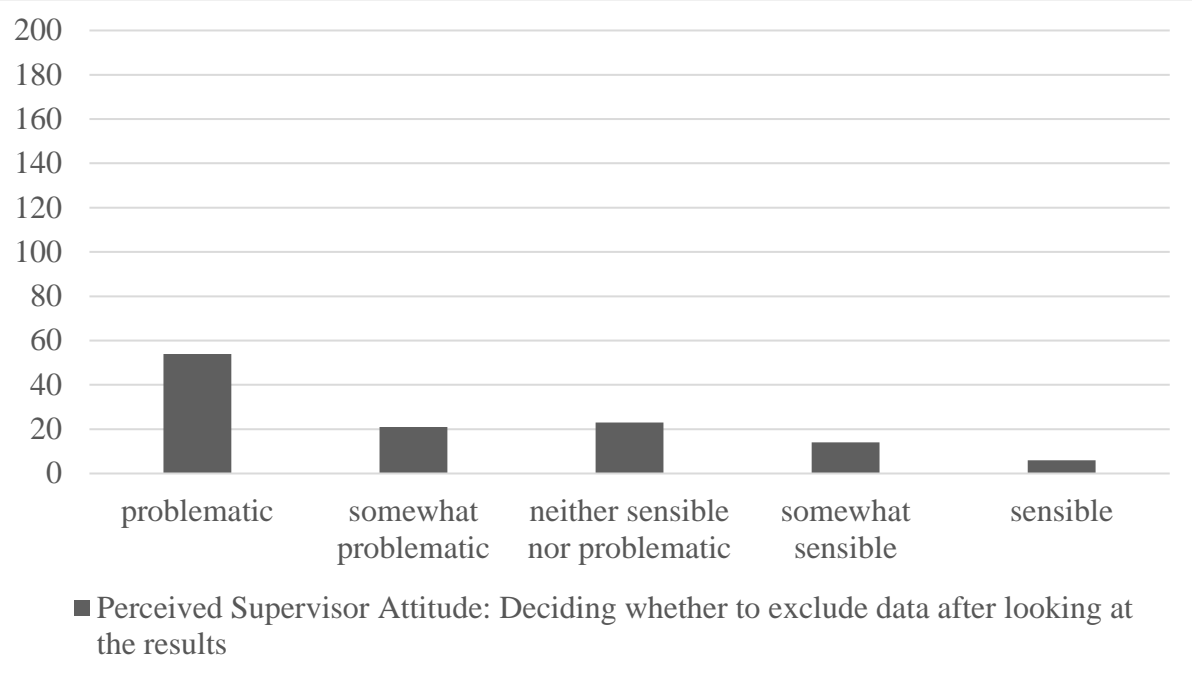

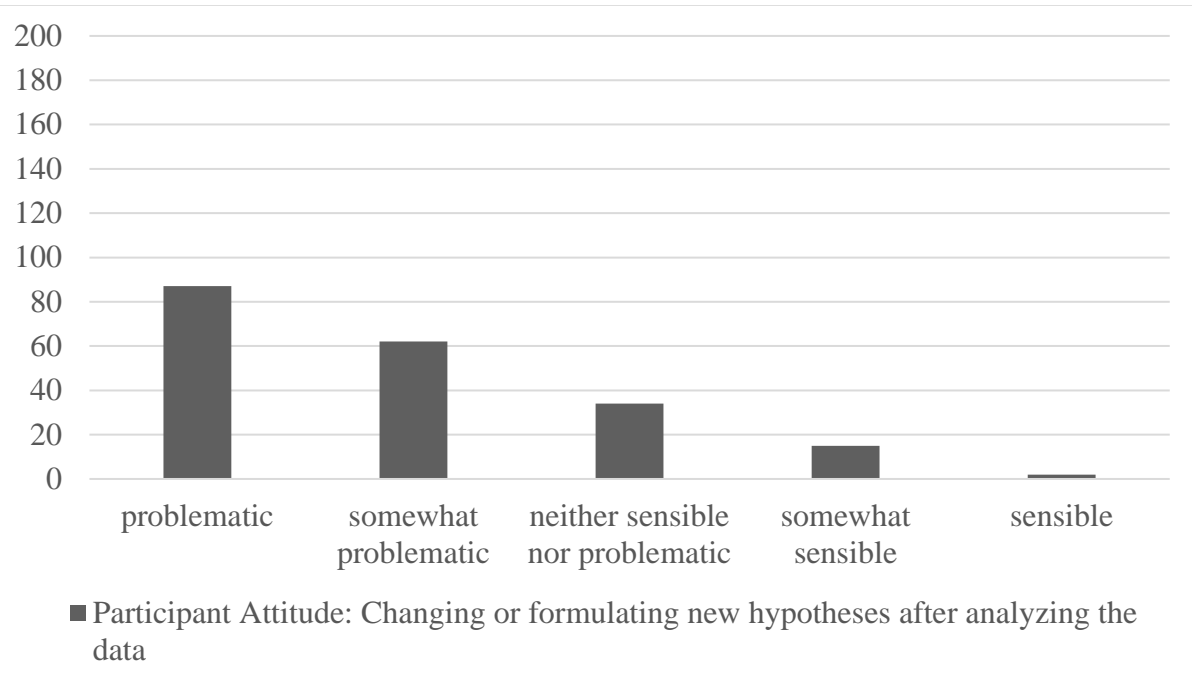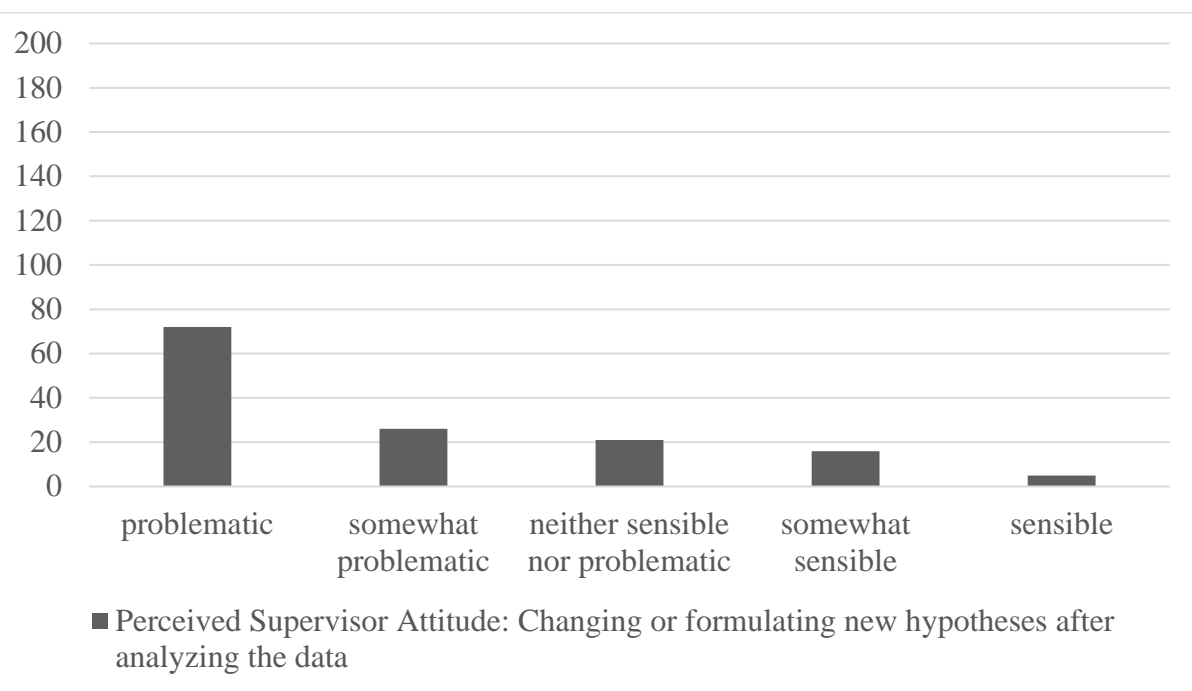

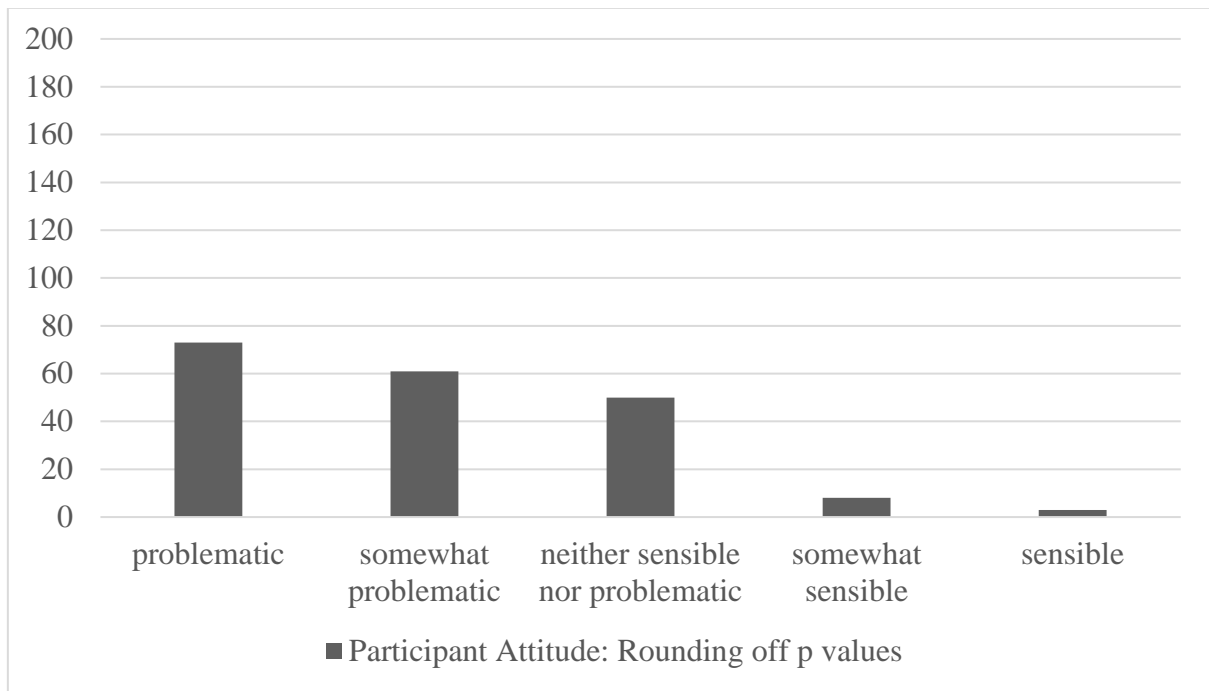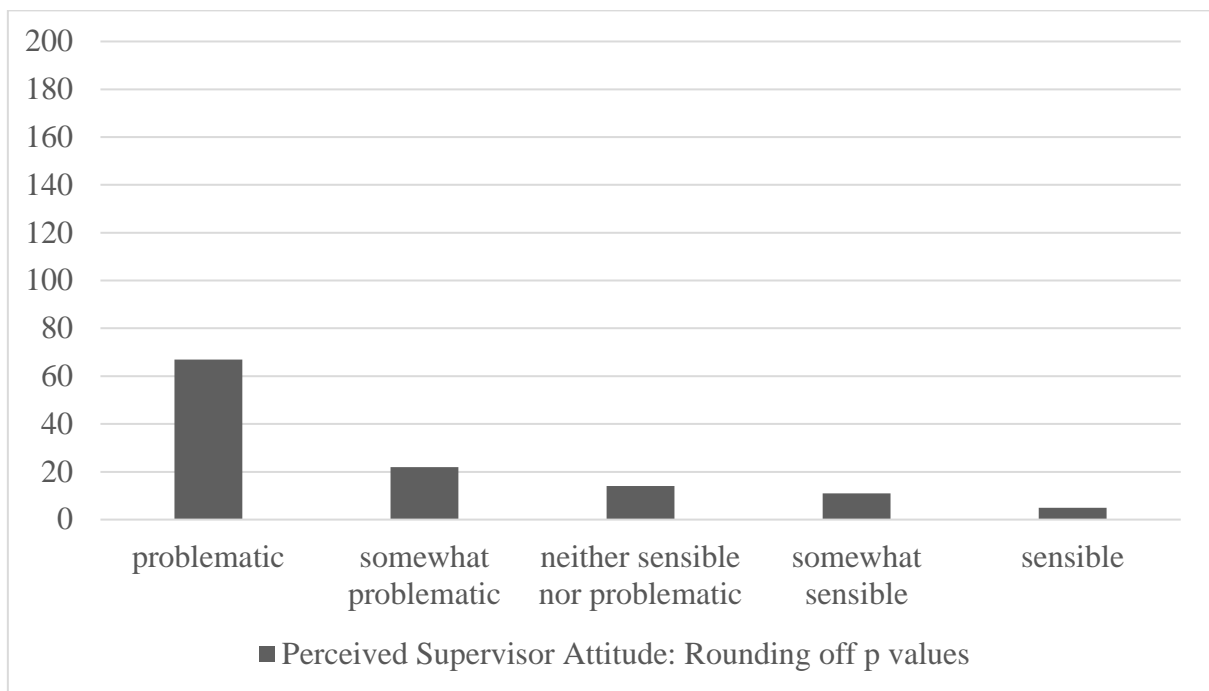

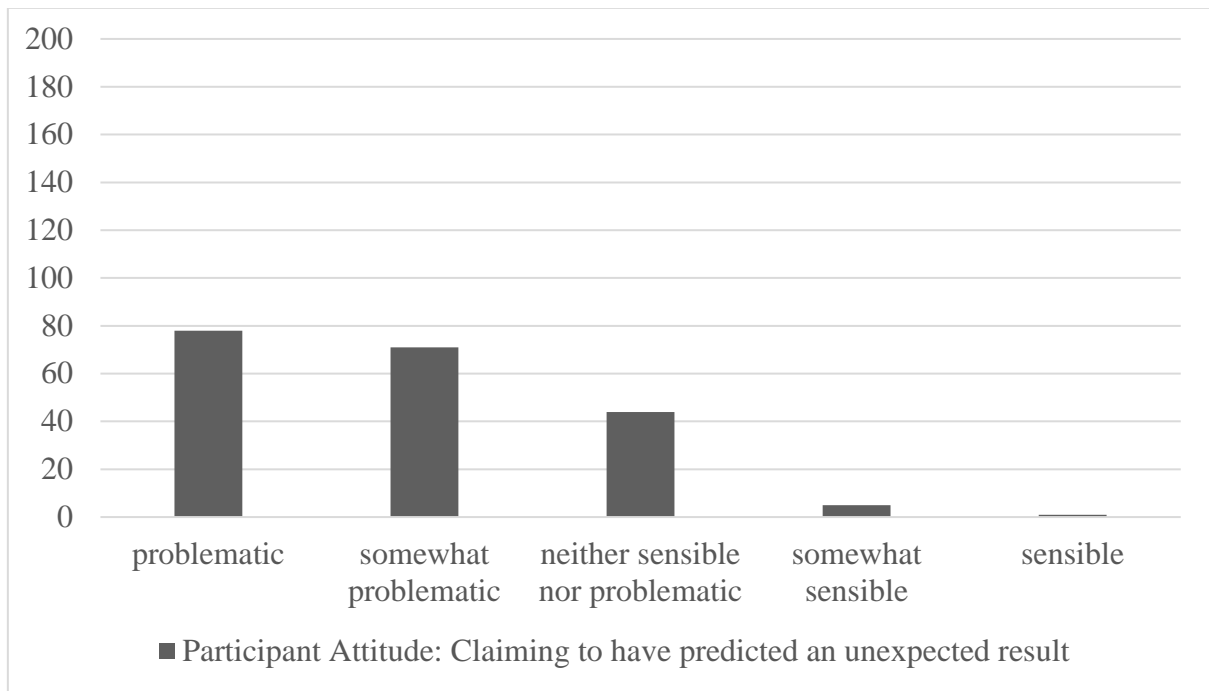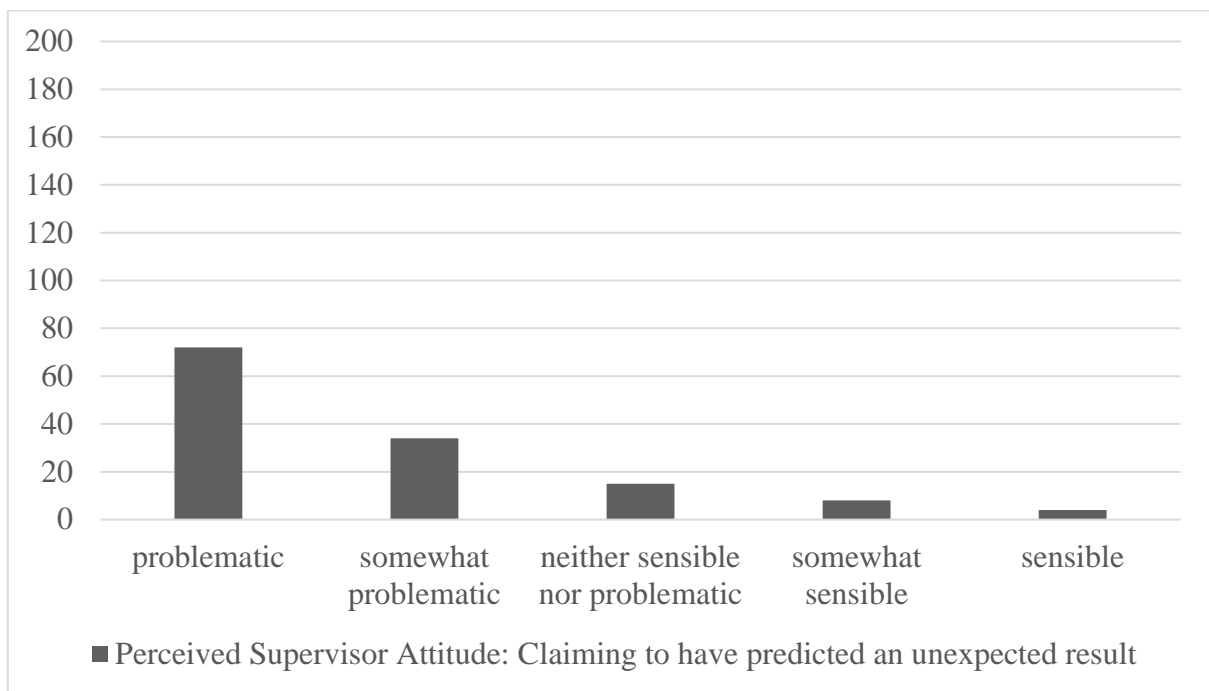

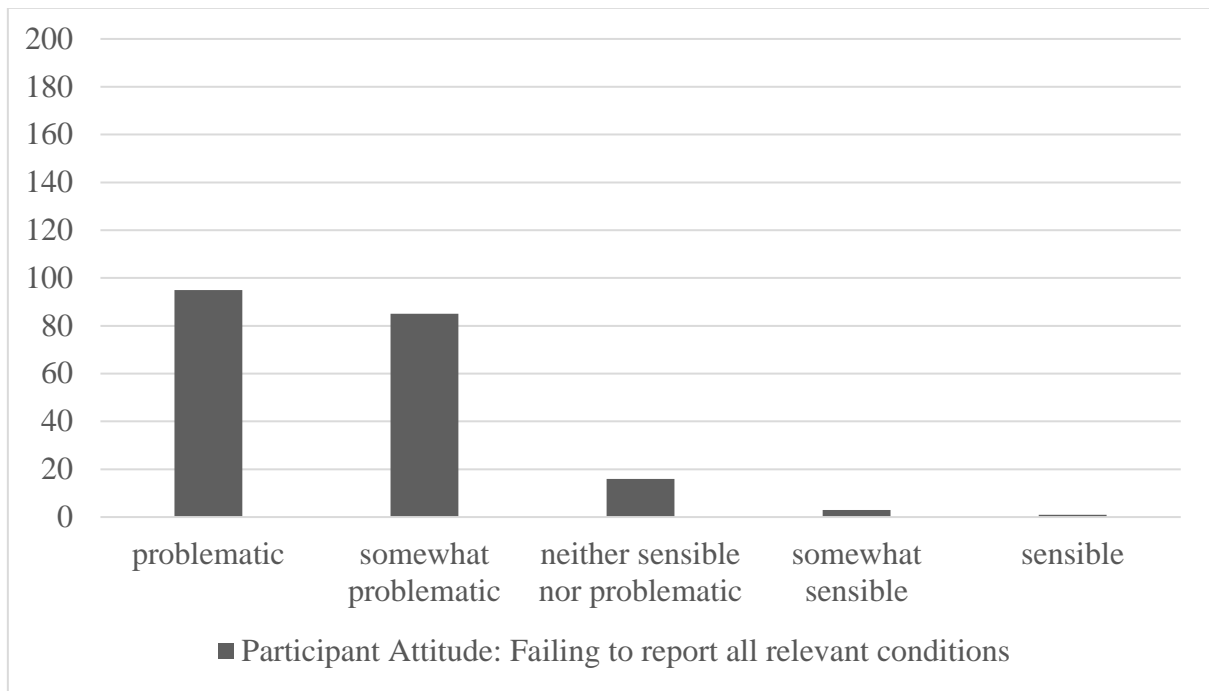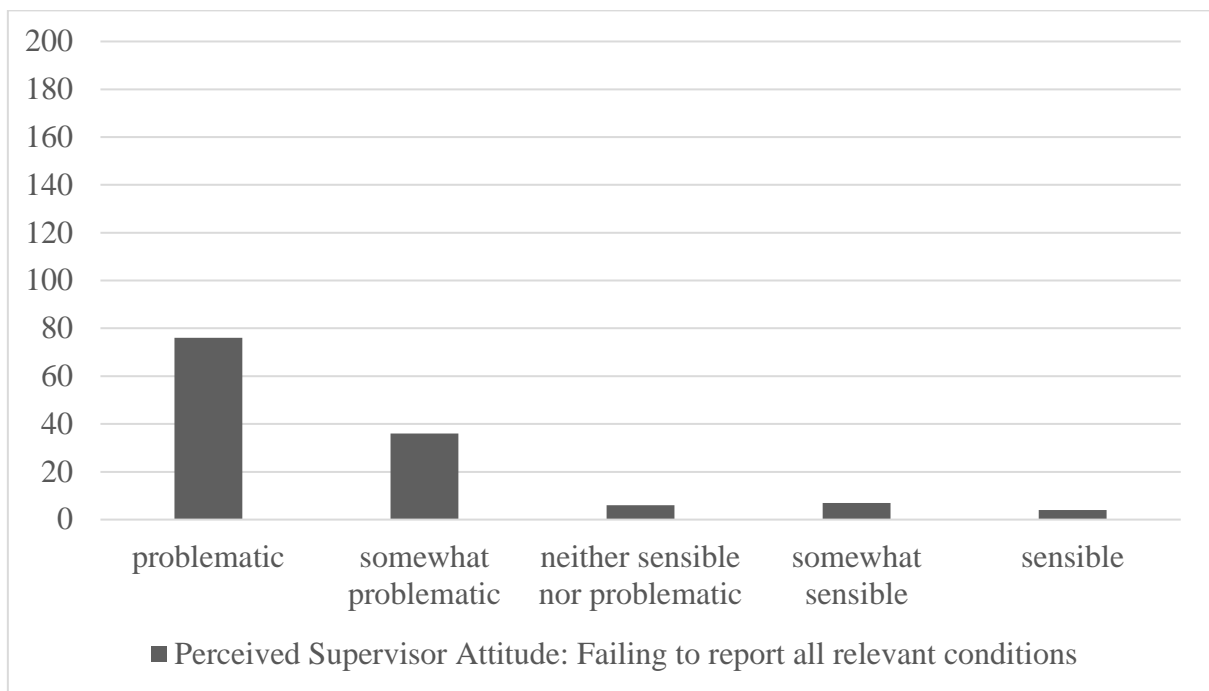

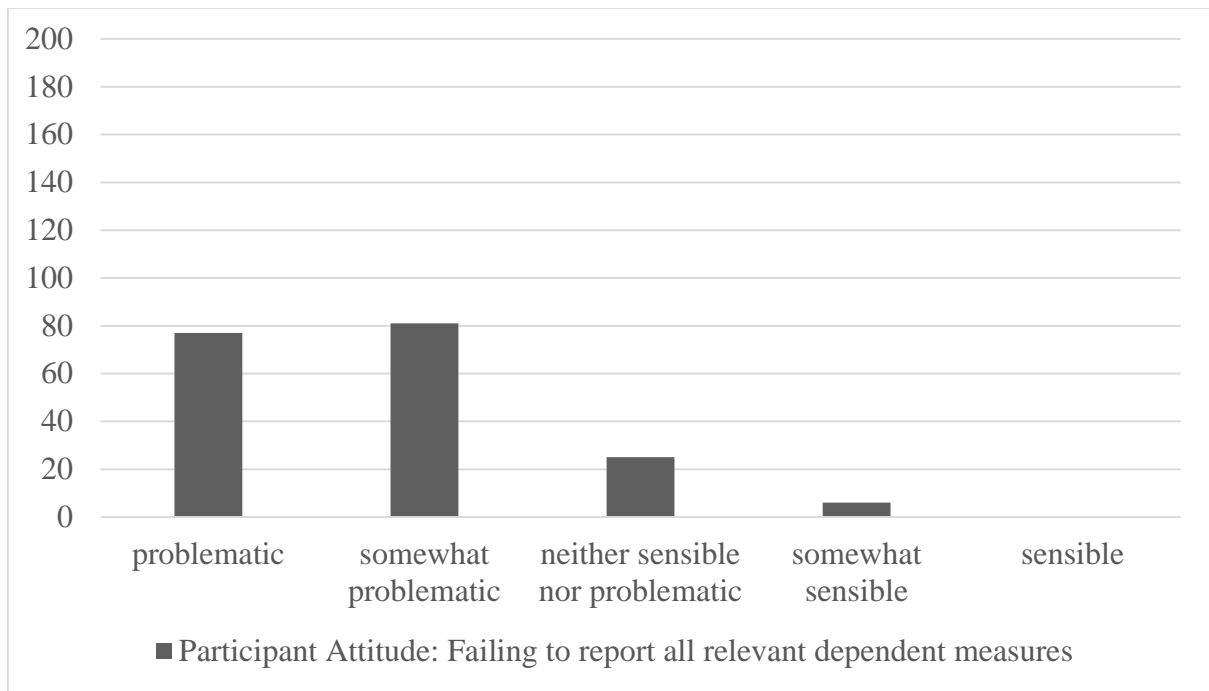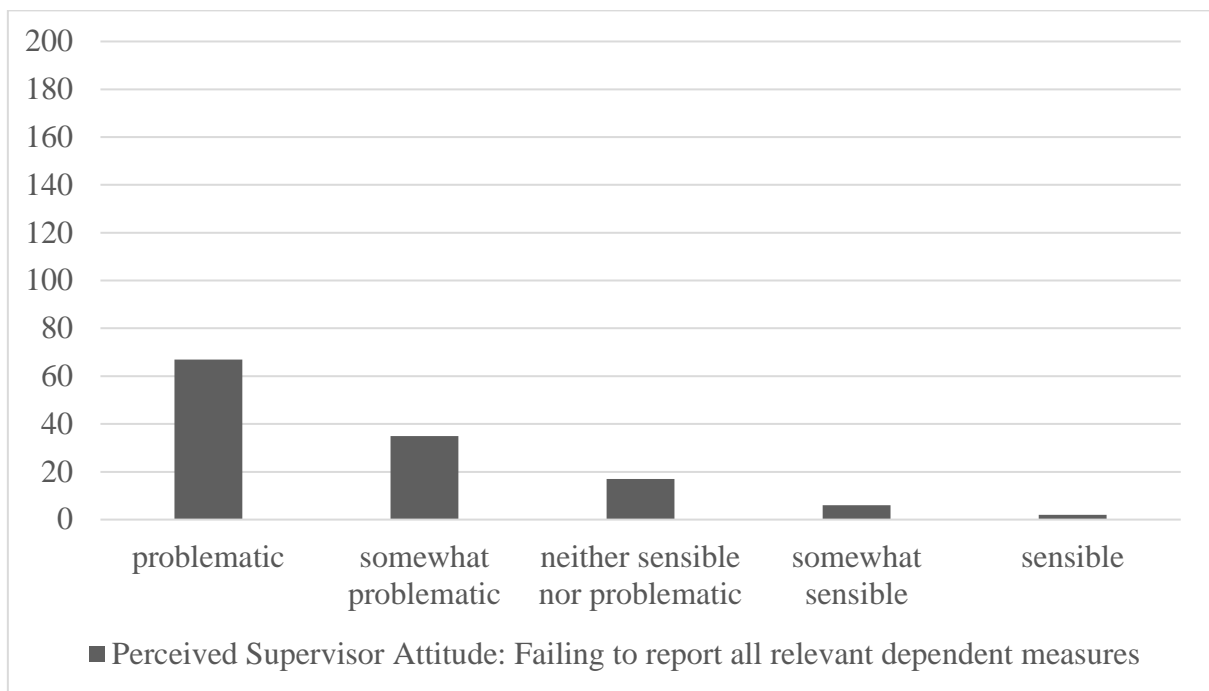

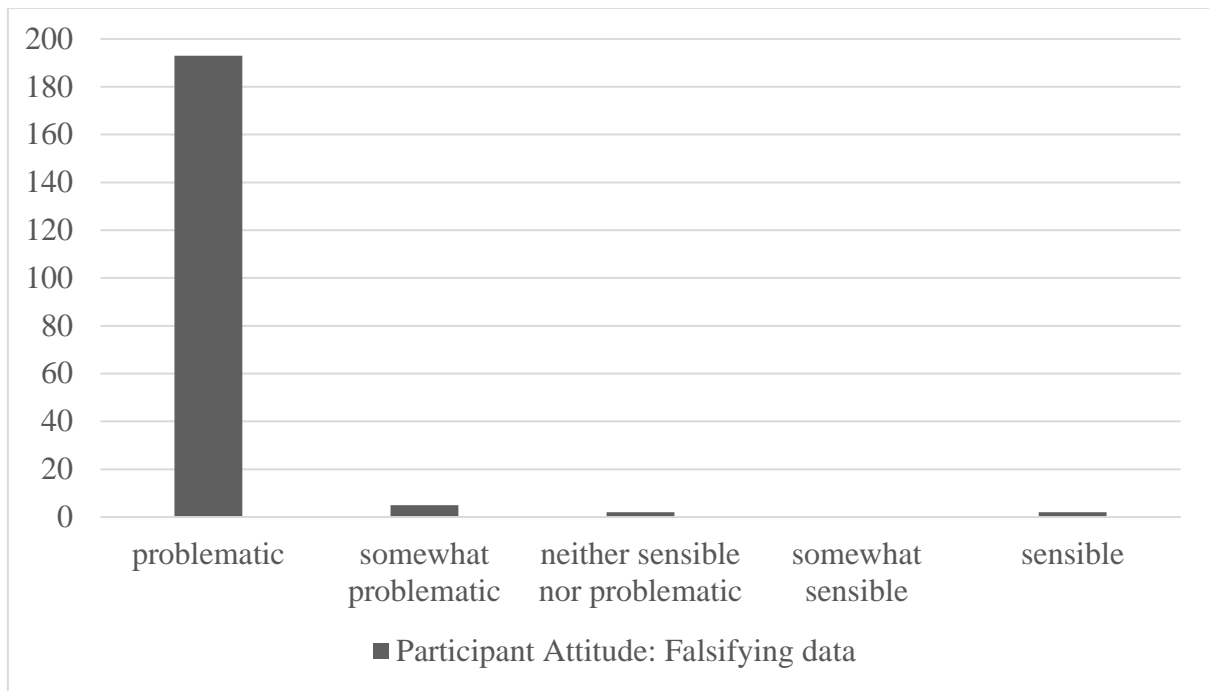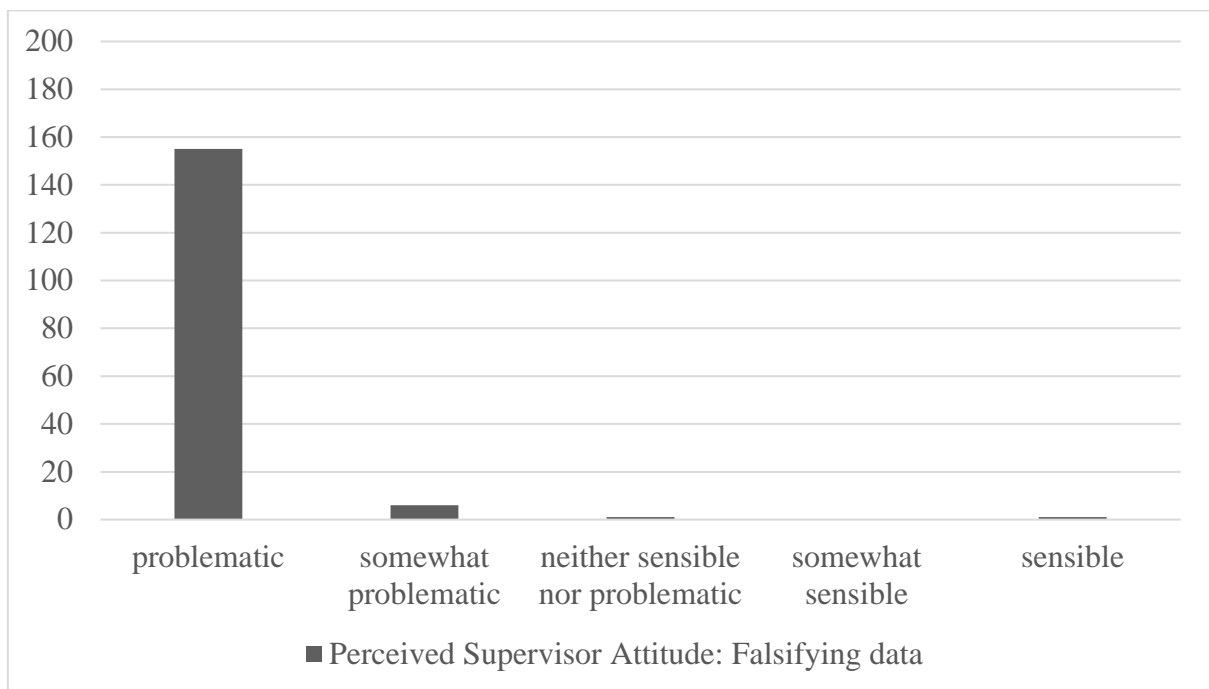

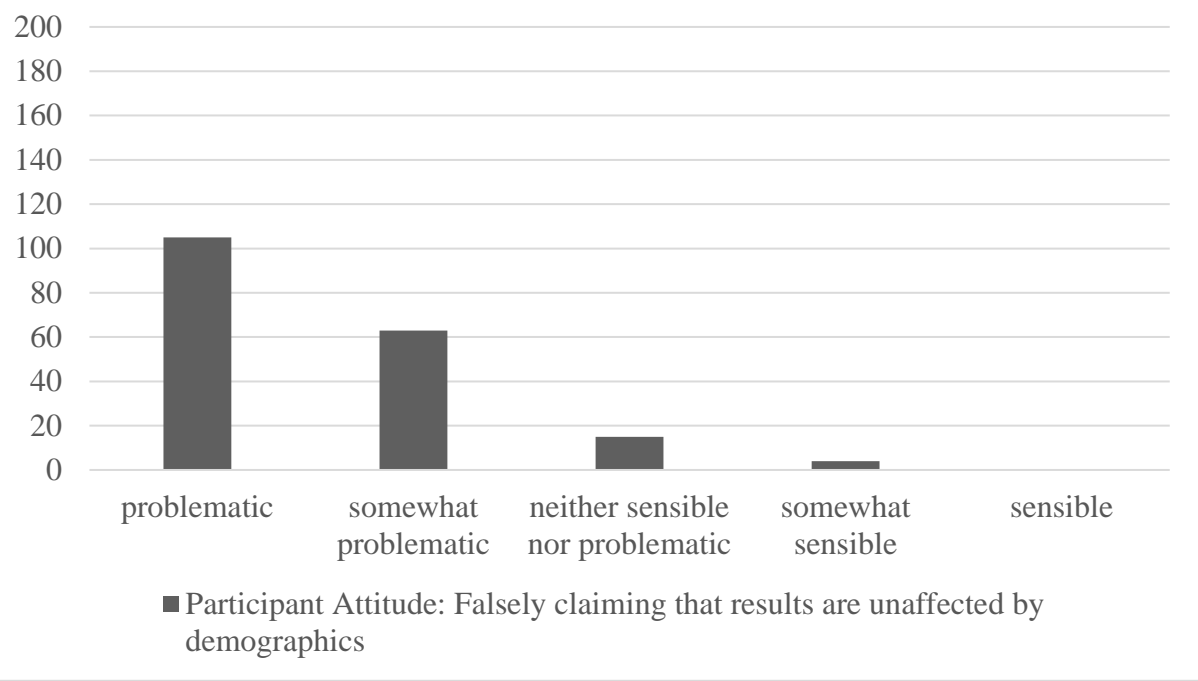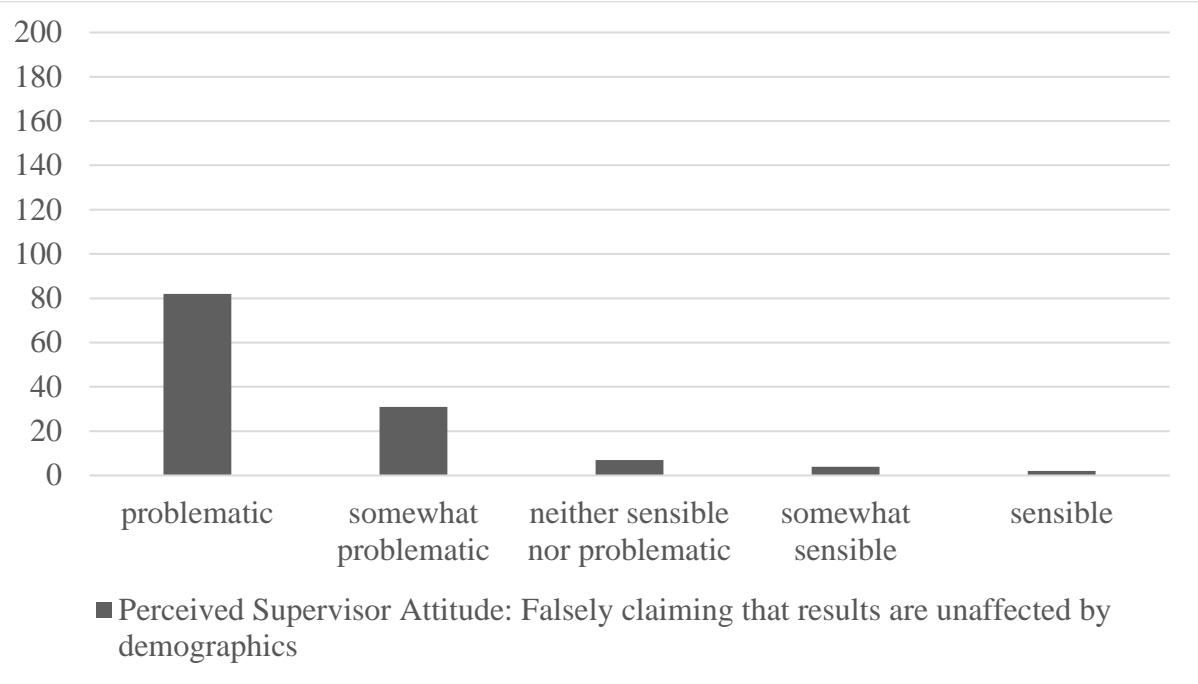

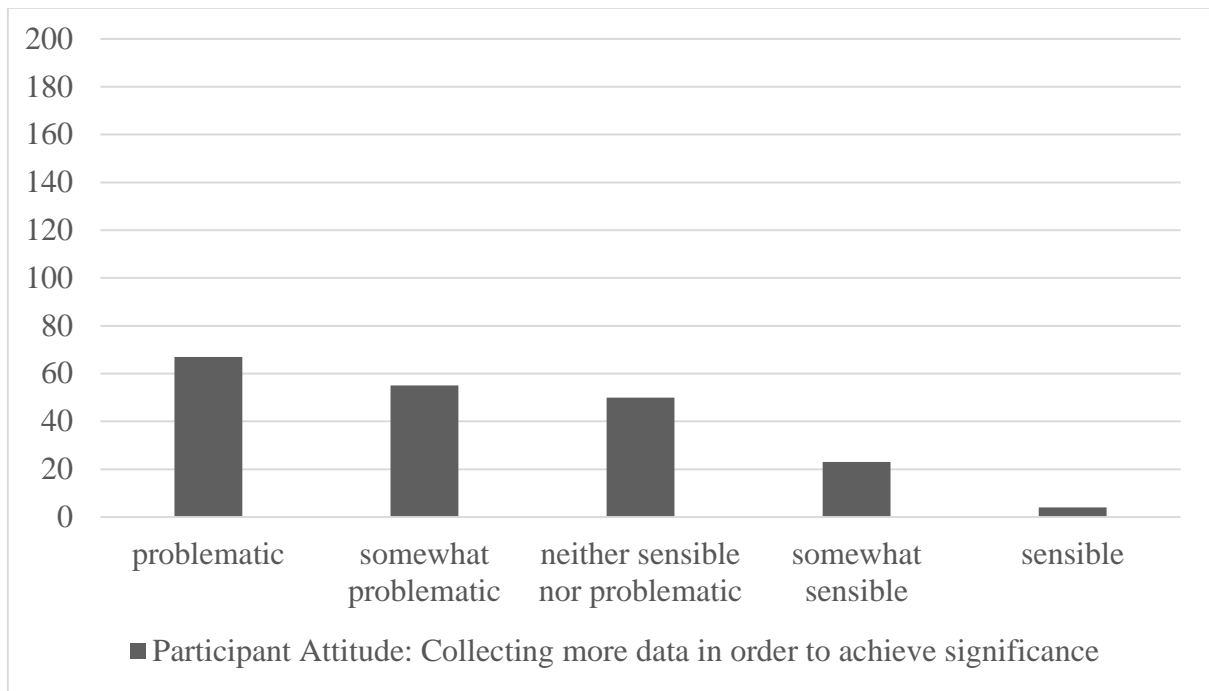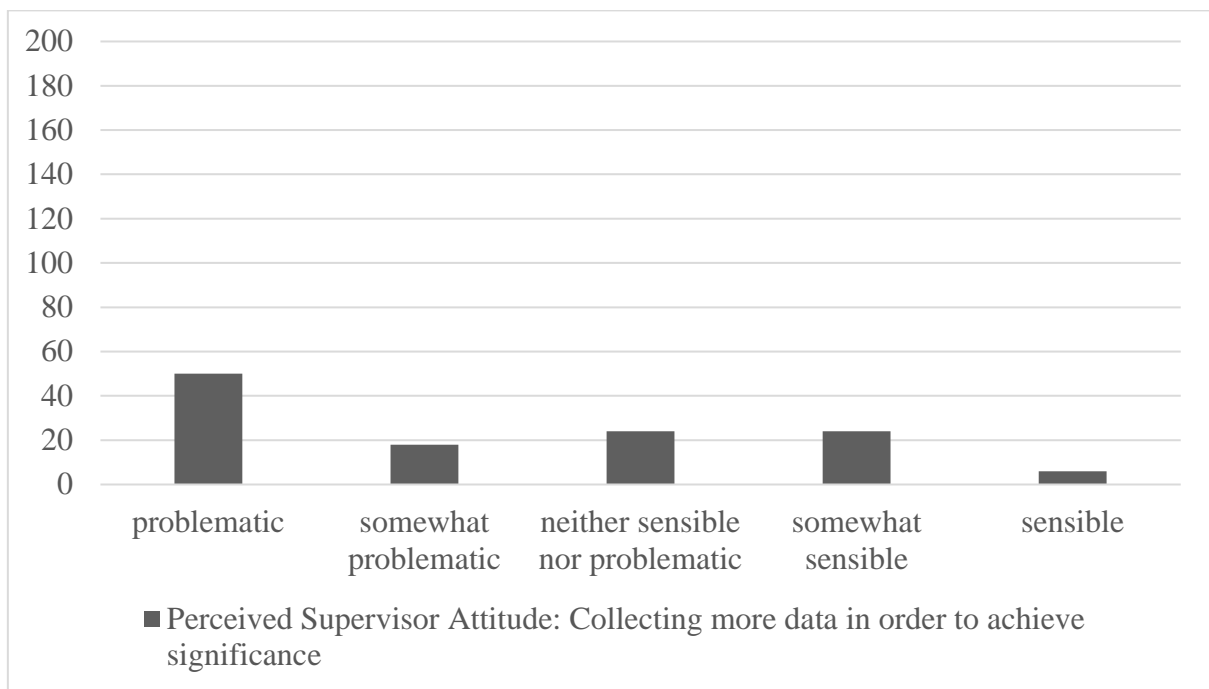

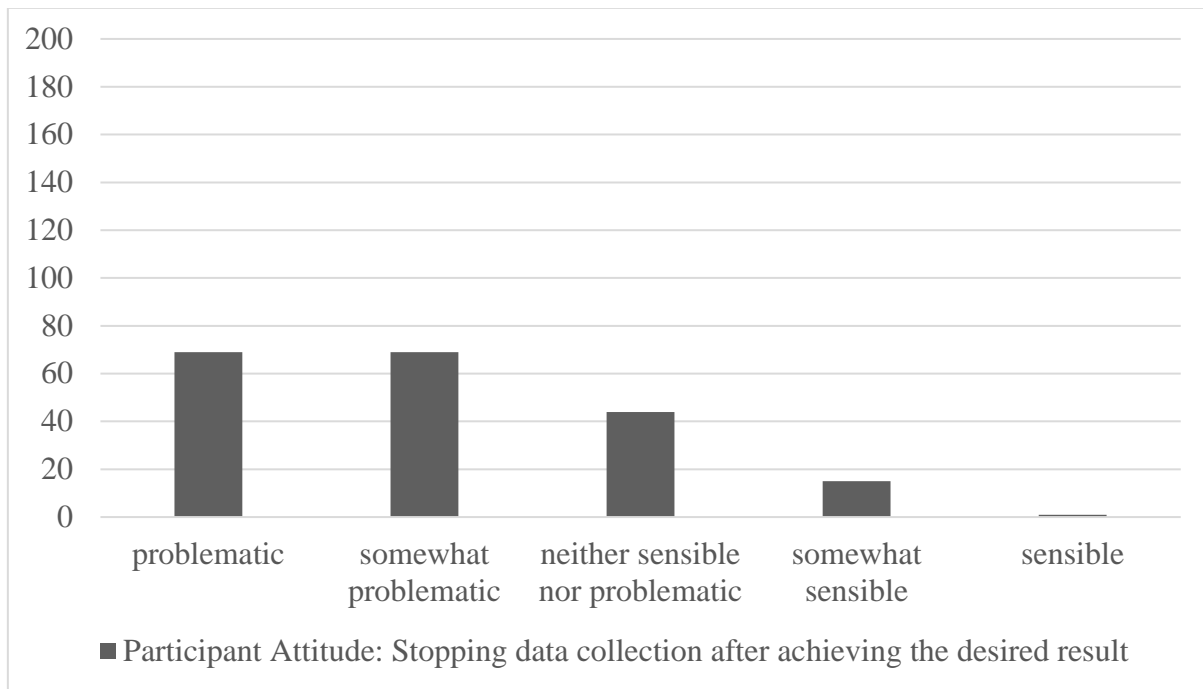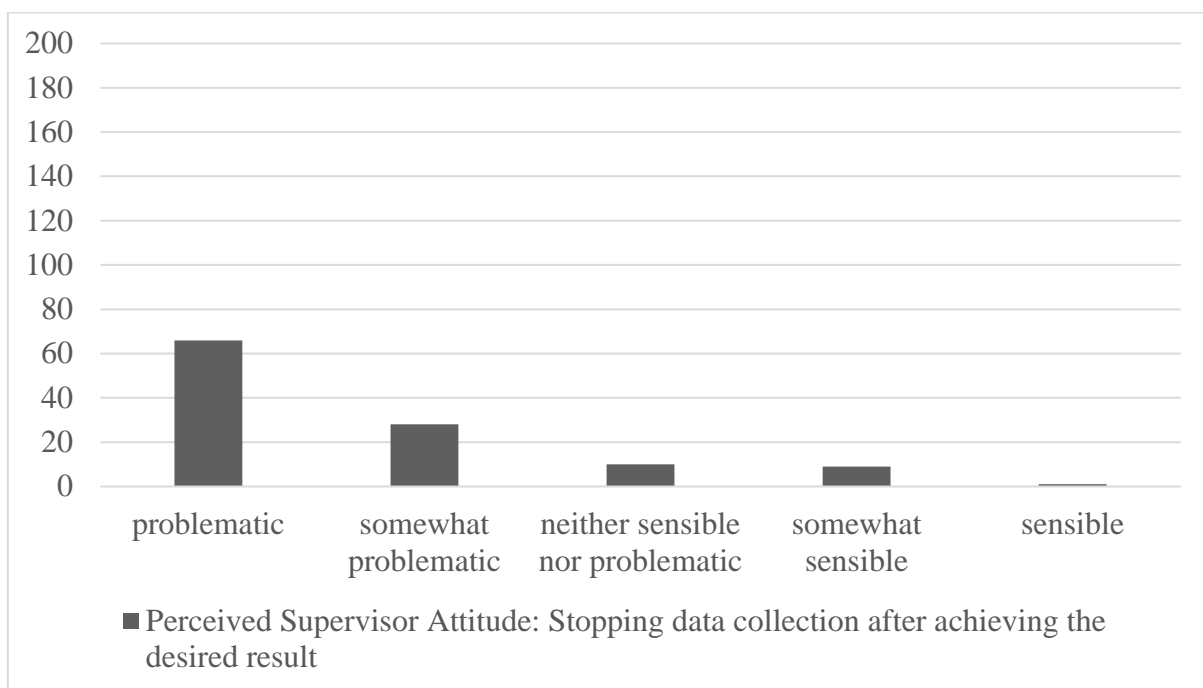

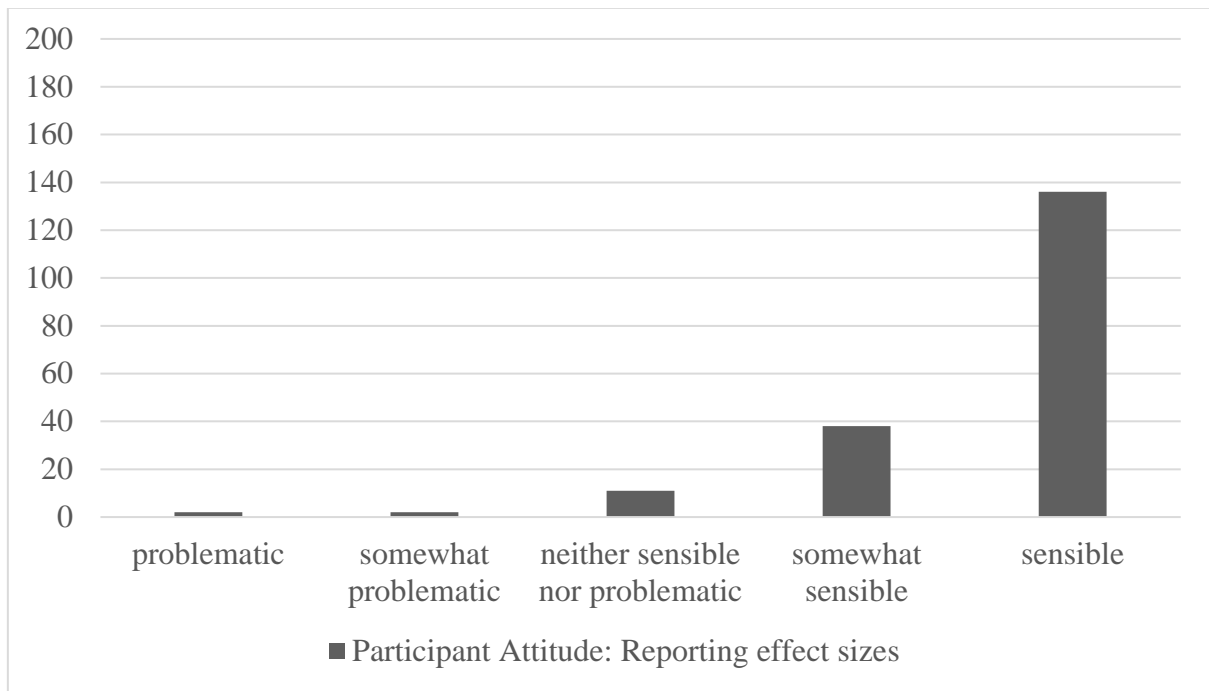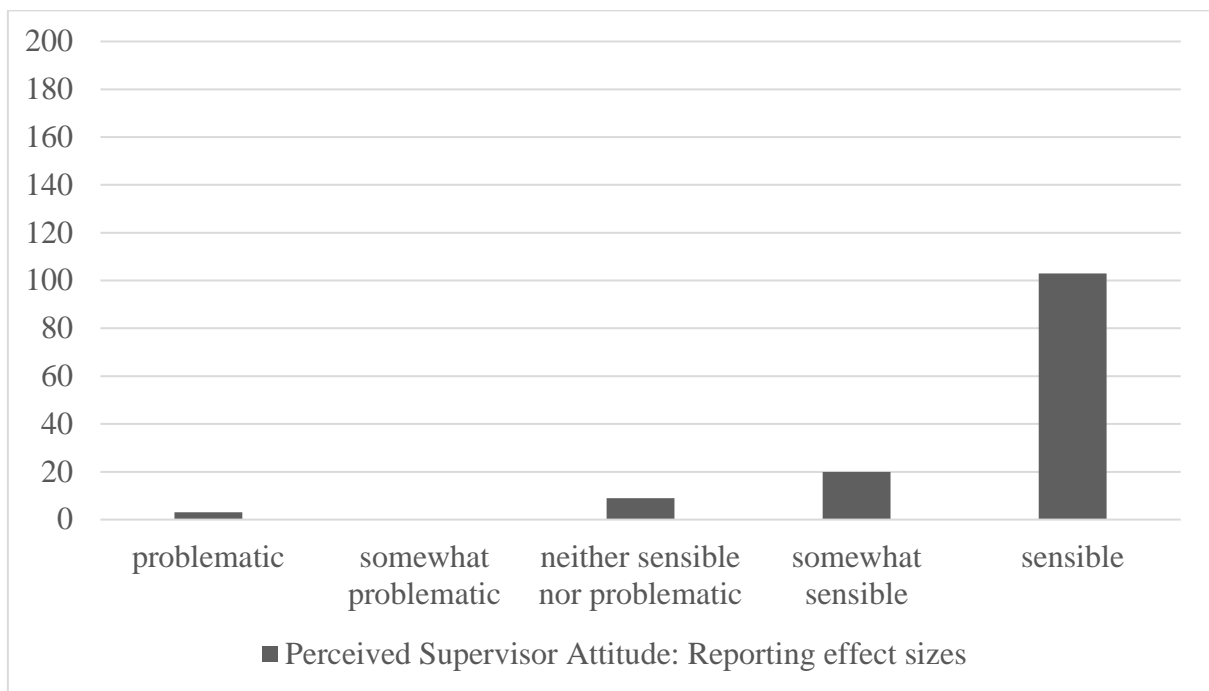

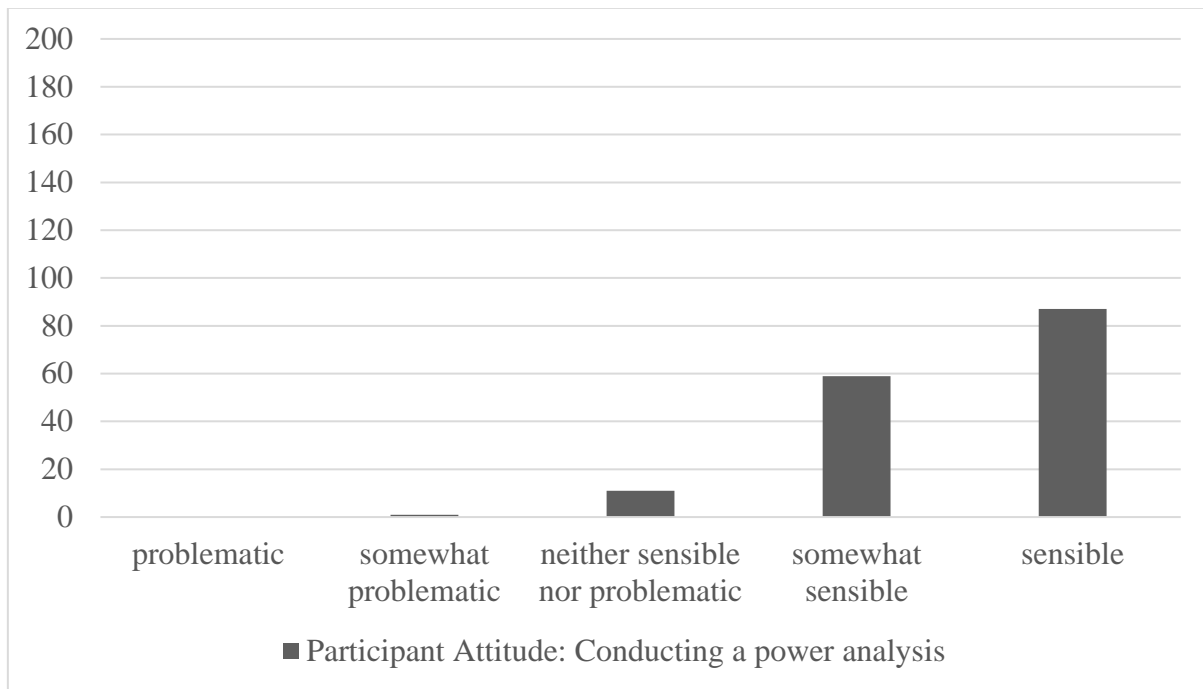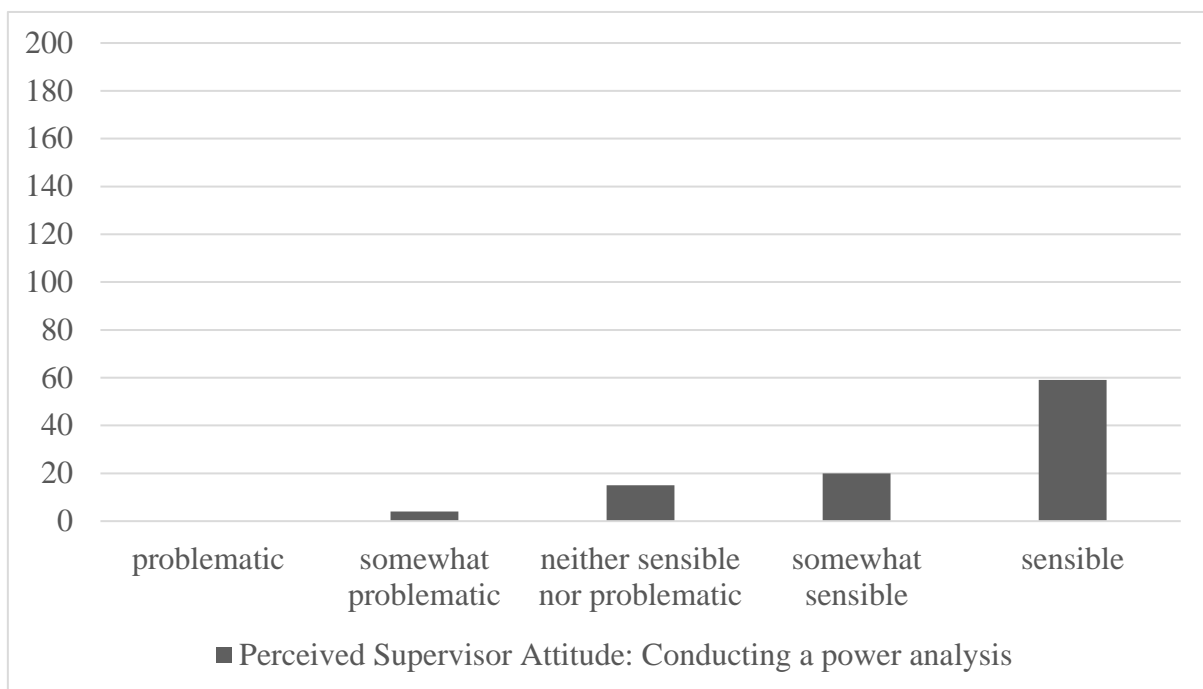

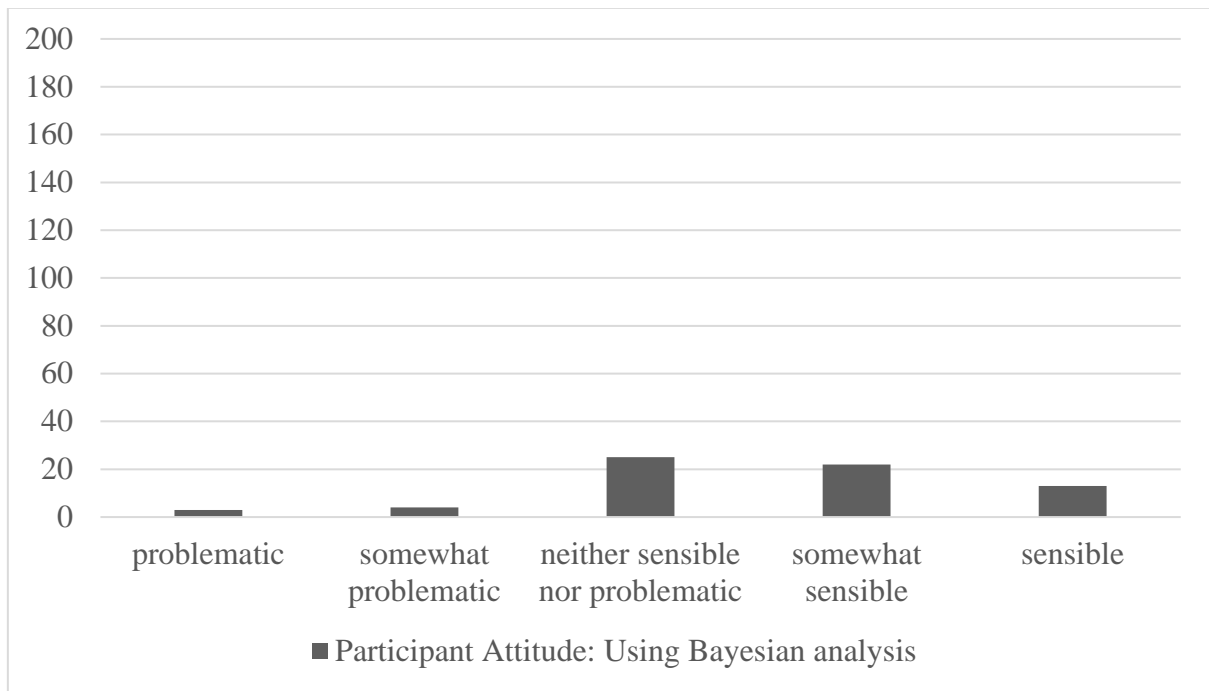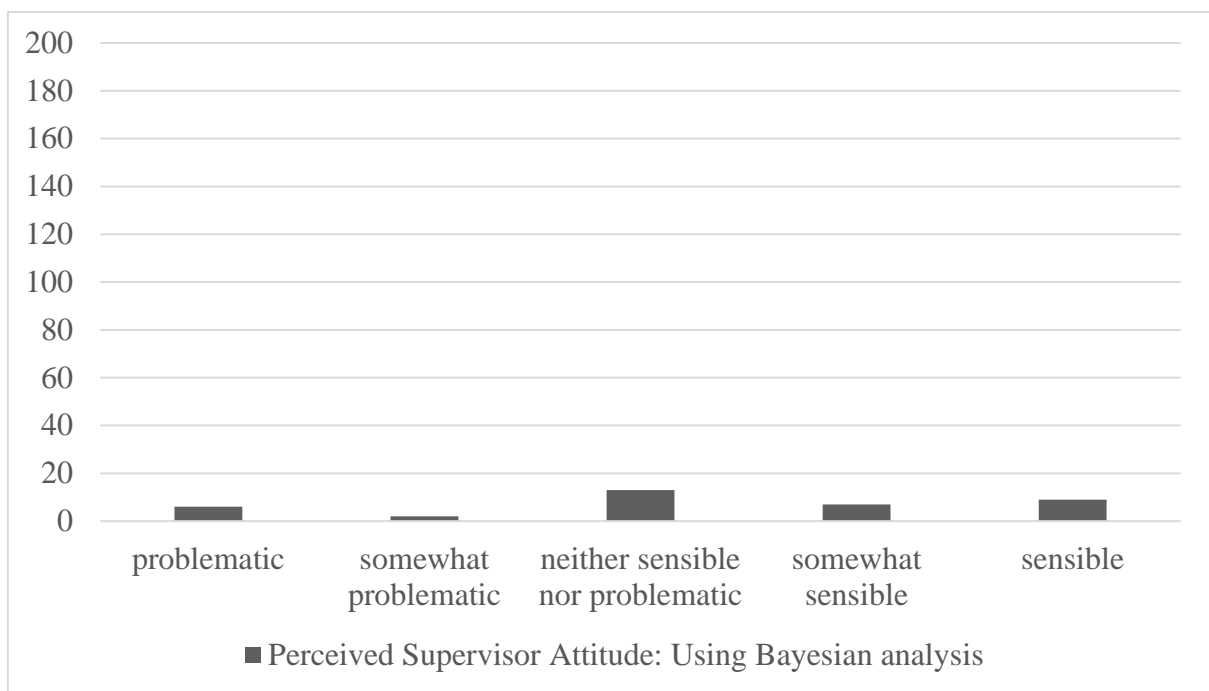

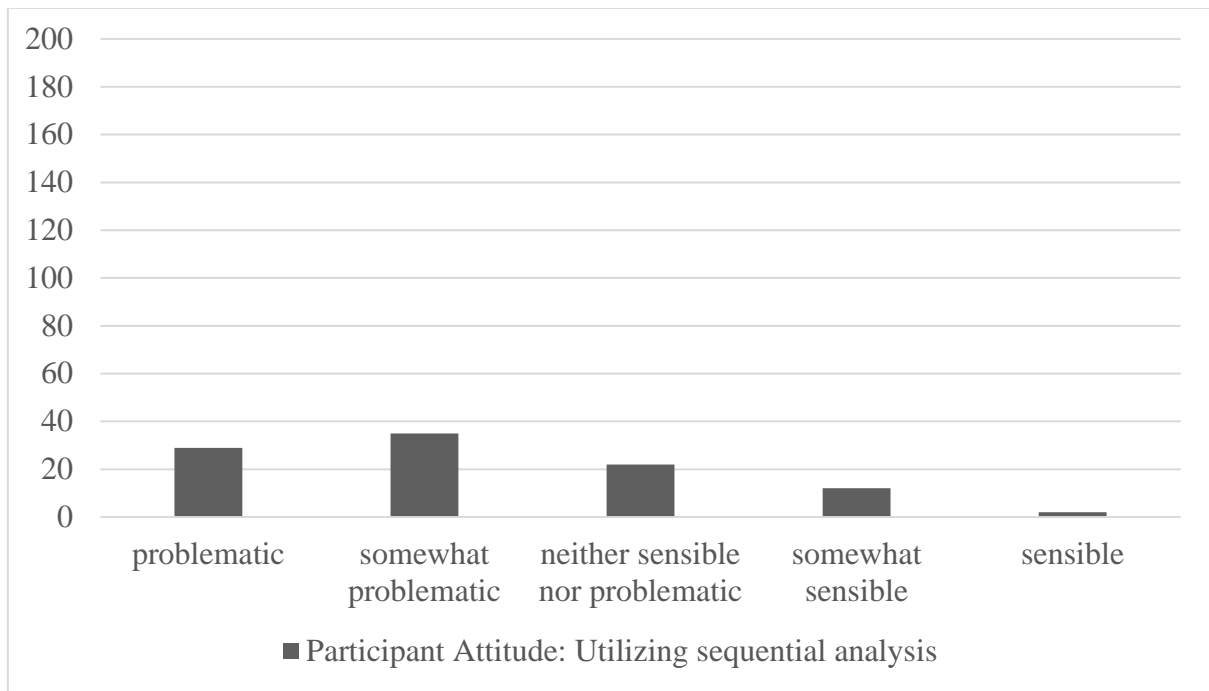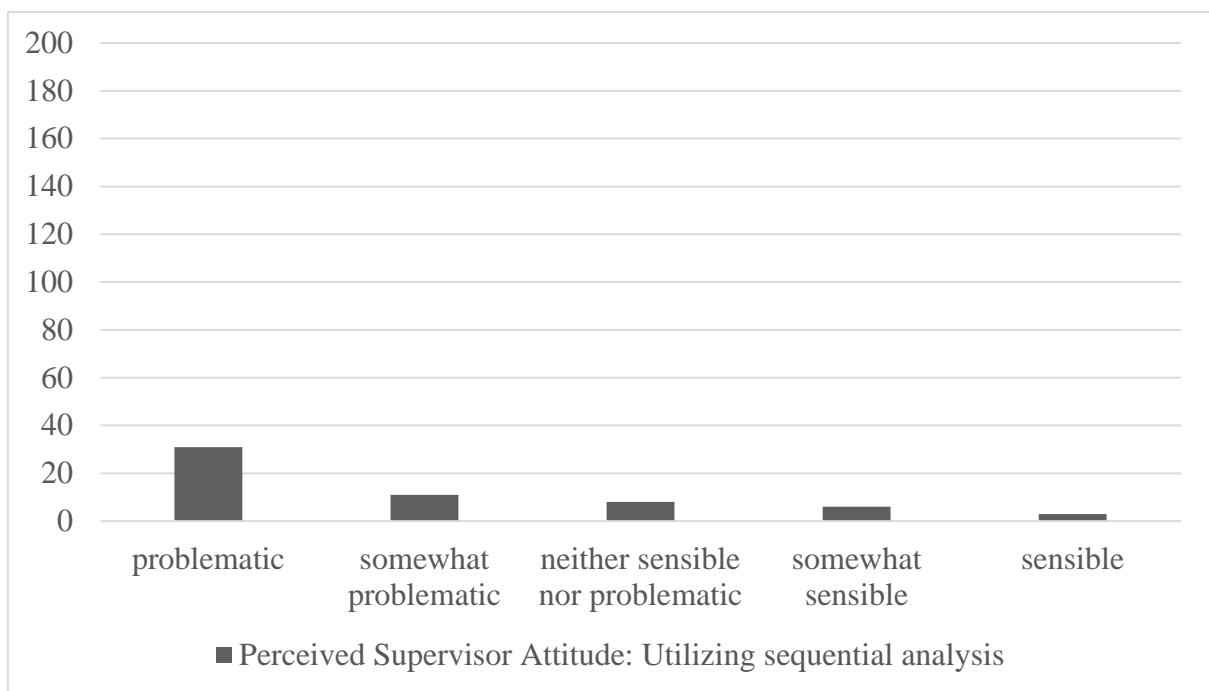

Supplement: S1 Bar Charts — (PDF) [file pone.0203470.s004.pdf]
