# Supplementary material for: Questionable research practices in student final theses – Prevalence, attitudes, and the role of the supervisor’s perceived attitudes
Source: PLoS One. 2018 Aug 30;13(8):e0203470. doi: 10.1371/journal.pone.0203470 (PMC6117074; doi:10.1371/journal.pone.0203470)
Supplement: S1 Exploratory Analysis — (PDF) [file pone.0203470.s005.pdf]

Analysis A

Regression models were calculated using mean-standardized predictors. The predictor was motivation and the moderator was belief that good science leads to significant results for the first model and belief that the supervisor rewards significant results for the second. Each model pair was applied to reporting and analysis QRPs and study design QRPs.

For reporting and analysis QRPs, the first model achieved significance, $F(3,175) = 5.84$, $p < .001$, Adj. $R^2 = .075$. Motivation achieved significance ($B = -.049$, $SE = .012$, $p < .001$), but no other predictors did (all $p \geq .33$). The second model also achieved significance, $F(3,175) = 7.45$, $p < .001$, Adj. $R^2 = .098$, producing a main effect of motivation ($B = -.049$, $SE = .012$, $p < .001$) and a marginally significant interaction of motivation and belief that the supervisor rewards significant results ($B = -.030$, $SE = .017$, $p = .081$). Opposite to expectations, belief that a significant result is required for a good grade actually *increases* the protective effect of motivation against self-reported QRP use.

For study design QRPs, the first model achieved significance, $F(3,170) = 2.98$, $p = .033$, Adj. $R^2 = .033$. Motivation achieved significance ($B = -.023$, $SE = .010$, $p = .018$), but no other predictors did (all $p \geq .241$). For the second model, the same effects obtained (motivation: $B = -.025$, $SE = .009$, $p = .009$; all other $p \geq .255$). It is worth noting, however, that the interaction effect was positive for both of these models, indicating a descriptive trend in the expected direction.
